# Evolution and development of complex floral displays

**DOI:** 10.1242/dev.203027

**Published:** 2024-11-05

**Authors:** Farahnoz N. Khojayori, Udhaya Ponraj, Kristina Buch, Yi Zhao, Humberto Herrera-Ubaldo, Beverley J. Glover

**Affiliations:** Department of Plant Sciences, University of Cambridge, Downing Street, Cambridge CB2 3EA, UK

**Keywords:** Complex flowers, Evo-devo, Plant evolution, Pollinator shifts

## Abstract

Flowering plants – angiosperms – display an astounding diversity of floral features, which have evolved in response to animal pollination and have resulted in the most species-rich plant clade. Combinations of macroscale (e.g. colour, symmetry, organ number) and microscale (e.g. cell type, tissue patterning) features often lead to highly elaborate floral displays. Most studies have focused on model species with simple floral displays to uncover the genetic and evolutionary mechanisms involved in flower evolution, yet few studies have focused on complex floral displays. Here, we review current knowledge on the development and evolution of complex floral displays. We review gene regulatory networks involved in four developmental pathways contributing to overall floral display (inflorescence architecture, organ identity, flower symmetry and flower colour) in classical plant models. We then discuss how evolutionary modification of one or more of these pathways has resulted in the production of a range of complex floral displays. Finally, we explore modular systems in which multiple pathways have been modified simultaneously, generating the most elaborate floral displays.

## Teaser

**Summary:** There are over 300,000 species of flowering plants, with enormous variation in their floral display. This Review explores the development of this diversity, based on our understanding from model systems.

## Introduction

Since Laibach's description of *Arabidopsis thaliana* L. Heynh in 1943 as a small plant with few chromosomes, short life cycle and abundant natural variation, the plant has become a giant in the field of plant biology (Meyerowitz, 2001). *Arabidopsis* was the first plant genome sequenced, and currently has the best annotated genome in the plant kingdom (The Arabidopsis Genome Initiative, 2000; Bevan et al., 2001). Nearly all current understanding of plant genes, RNAs and proteins is best known in *Arabidopsis*; this understanding has subsequently been extended by other established models, which are often far behind *Arabidopsis*, or by non-model species (Woodward and Bartel, 2018). However, *Arabidopsis* is an unusual plant to represent over 350,000 species of angiosperms (Freiberg et al., 2020; Qian et al., 2022). It is part of a medium-sized family (Brassicaceae) within core eudicots and its flowers have the uncommon number of four petals, which lack colourful pigmentation, on a raceme (a simple indeterminate inflorescence; see Glossary, Box 1). Its flower morphology alone fails to capture the diversity in organ types, floral colour, floral symmetry and other elaborate traits often seen in angiosperms, especially those among non-eudicot lineages. A second model system for the study of flower development, *Antirrhinum majus* L., illustrates some of the elaborate floral traits lacking in *Arabidopsis*. Concomitant studies in both model systems have provided an in-depth overview of flower development, following the differentiation of meristematic (see Glossary, Box 1) cells into elaborate organs with unique identities.

**Box 1. Glossary**

**(unless otherwise specified, definitions adapted from: Weberling, 1989; Evert and Eichorn, 2012)**

**Actinomorphic:** Used to describe a flower that is radially symmetric, with multiple planes of symmetry.

**Androdioecy:** A plant reproductive strategy where bisexual and male flowers occur on separate individuals (Renner, 2014).

**Andromonoecy:** A plant reproductive strategy where bisexual and male flowers occur on the same individual (Renner, 2014).

**Anthesis:** The flowering period of a plant from opening until seed set.

**Axil:** The upper angle between a twig or leaf and the stem from which it grows.

**Bract:** A reduced leaf. This is commonly a subtending leaf in inflorescences.

**Determinate inflorescence:** An inflorescence type where the axis of growth ends in a flower.

**Gynodioecy:** A plant reproductive strategy where bisexual and female flowers occur on separate individuals.

**Gynomonoecy:** A plant reproductive strategy where bisexual and female flowers occur on the same individual.

**Indeterminate inflorescence:** An inflorescence type where the axis of growth never produces a terminal flower.

**Inflorescence:** A structure composed of modified leaves, stems and flowers representing the reproductive life stage of angiosperms.

**Meristem:** A tissue region within a plant composed of pluripotent cells.

**Monoecy:** A plant reproductive strategy where anthers and carpels are produced on separate flowers of the same individual.

**Perianth:** A collective term for the sterile flower organs (the sepals and petals).

**Perfect flower:** A flower that has both stamens and carpels (i.e. a hermaphroditic or bisexual flower).

**Staminodes:** Sterile stamens.

**Tepal:** One of the outer parts of the flower (perianth). This term is used when there is no clear difference between sepals and petals.

**Terminal flower:** A flower formed at the apex; terminates the principal axis or racemose branches.

**Whorl:** A circular arrangement of organs around an axis of a plant.

**Zygomorphic:** Used to describe a flower that is bilaterally symmetric, with one plane of symmetry.

Bateman et al. (2006) define a flower as a ‘determinate axis bearing megasporangia that are surrounded by microsporangia and are collectively subtended by at least one sterile laminar organ’ (Bateman et al., 2006). This definition encompasses the pivotal innovation of the angiosperms in bearing both male (i.e. microsporangia) and female (i.e. megasporangia) reproductive structures on the same axis or shoot. These reproductive structures are then surrounded by sterile modified leaves, typically composed of showy sepals and petals, as illustrated in *Antirrhinum*. A flower, as a reproductive unit, may appear on an axis alone or develop as a repeated unit on a larger reproductive structure, called an inflorescence (see Glossary, Box 1). Arrangement, number, pattern, identity, symmetry and colour of the individual organs of a flower, and of the flowers within the inflorescence, lead to infinite possible combinations of floral displays. We focus on four traits which lend complexity to angiosperm reproductive morphology: (1) inflorescence architecture, (2) organ identity, (3) flower symmetry and (4) flower colour. We first discuss known developmental pathways from *Arabidopsis* and *Antirrhinum* flowers that underpin these four structural traits. We then go on to explore how these developmental pathways have been modified to produce complex floral displays in other systems. While the sheer number of complex floral displays in angiosperms prevents us considering all examples, we have selected examples where studies have revealed modification of the known developmental pathways to generate novel phenotypes. A summary of all species discussed in the Review is shown in Table S1.

## Building a floral display: a model perspective

Studies performed in model species have revealed the gene regulatory networks (GRNs) underpinning four traits that lend complexity to floral displays (inflorescence architecture, organ identity, flower symmetry and flower colour). In this section, we summarise current knowledge of the mechanisms underpinning these traits in *Arabidopsis* and *Antirrhinum*.

### Inflorescence architecture

**Fig. 1.**
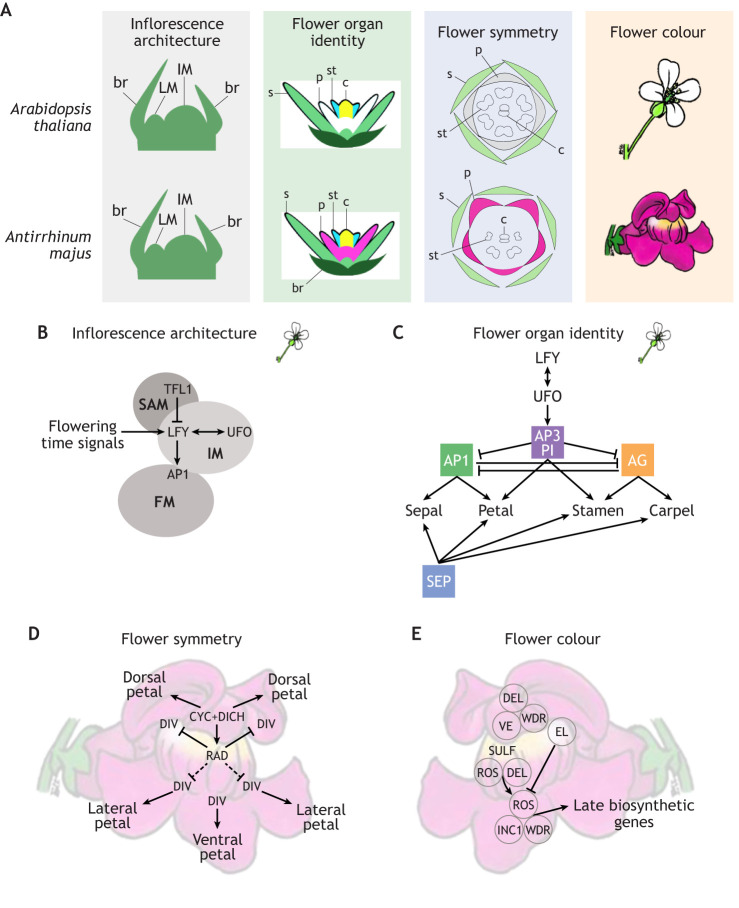
**Gene regulatory networks (GRNs) involved from flower initiation to anthesis in *Arabidopsis thaliana* and *Antirrhinum majus*.** (A) Schematics of four developmental milestones in flowering: establishment of inflorescence architecture, specification of floral organ identity, establishment of symmetry and establishment of flower colour. Inflorescence architecture is very similar between the two model species, as is the specification of floral organ identity, but differences emerge when floral symmetry and flower colour is established during the later stages of development. This results in the *Antirrhinum* flower being bilaterally symmetrical (zygomorphic) and coloured, while the *Arabidopsis* flower is radially symmetrical (actinomorphic) and unpigmented. br, bract; c, carpel; IM, inflorescence meristem; LM, lateral meristem; p, petal; s, sepal; st, stamen. (B-E) Simplified gene regulatory networks (GRNs) controlling four developmental pathways contributing to floral display. (B) The GRN controlling inflorescence architecture establishment in *Arabidopsis*, where the central regulator LFY is essential for activation of floral meristem identity. LFY is repressed in the inflorescence meristem by TFL1 to maintain indeterminacy of the inflorescence. Later in development, LFY and UFO interact to initiate expression of floral organ identity genes. AP1, APETALA 1; FM, floral meristem; IM, inflorescence meristem; LFY, LEAFY; SAM, shoot apical meristem; TFL1, TERMINAL FLOWER 1; UFO, UNUSUAL FLOWER ORGANS. (C) The GRN controlling floral organ identity specification in the *Arabidopsis* floral meristem, where the ABCE family transcription factors are expressed in overlapping domains, and in particular combinations, to specify the identity of different organs. A-function genes are green, B-function genes are purple, C-function genes are orange and E-function genes are blue. AG, AGAMOUS; AP1/3, APETALA 1/3; LFY, LEAFY; PI, PISTILLATA; SEP, SEPALLATA; UFO, UNUSUAL FLOWER ORGANS. (D) The GRN controlling establishment of flower symmetry in *Antirrhinum*, where the ventralising factor DIV is repressed in the dorsal parts of the flower by RAD. RAD itself is activated by dorsally expressed CYC. This ultimately results in the specification of dorsal, lateral and ventral petals. CYC, CYCLOIDEA; DICH, DICHOTOMA; DIV, DIVARICATA; RAD, RADIALIS. (E) The GRN controlling flower colour in *Antirrhinum* via anthocyanin production, where MBW complexes comprising different combinations of MYB and bHLH transcription factors and a scaffold protein (WDR) activate expression of genes encoding enzymes that drive red anthocyanin biosynthesis in different parts of the flower. ELUTA (EL), also a MYB protein, represses anthocyanin production in the petal lobes. A silencing RNA in the *SULFUREA* (*SULF*) locus results in yellow pigment being localised to the opening of the corolla tube. DEL, DELILA; INC1, INCOLORATA 1; ROS, ROSEA; VE, VENOSA; WDR, WD REPEAT PROTEINS.

**Fig. 2.**
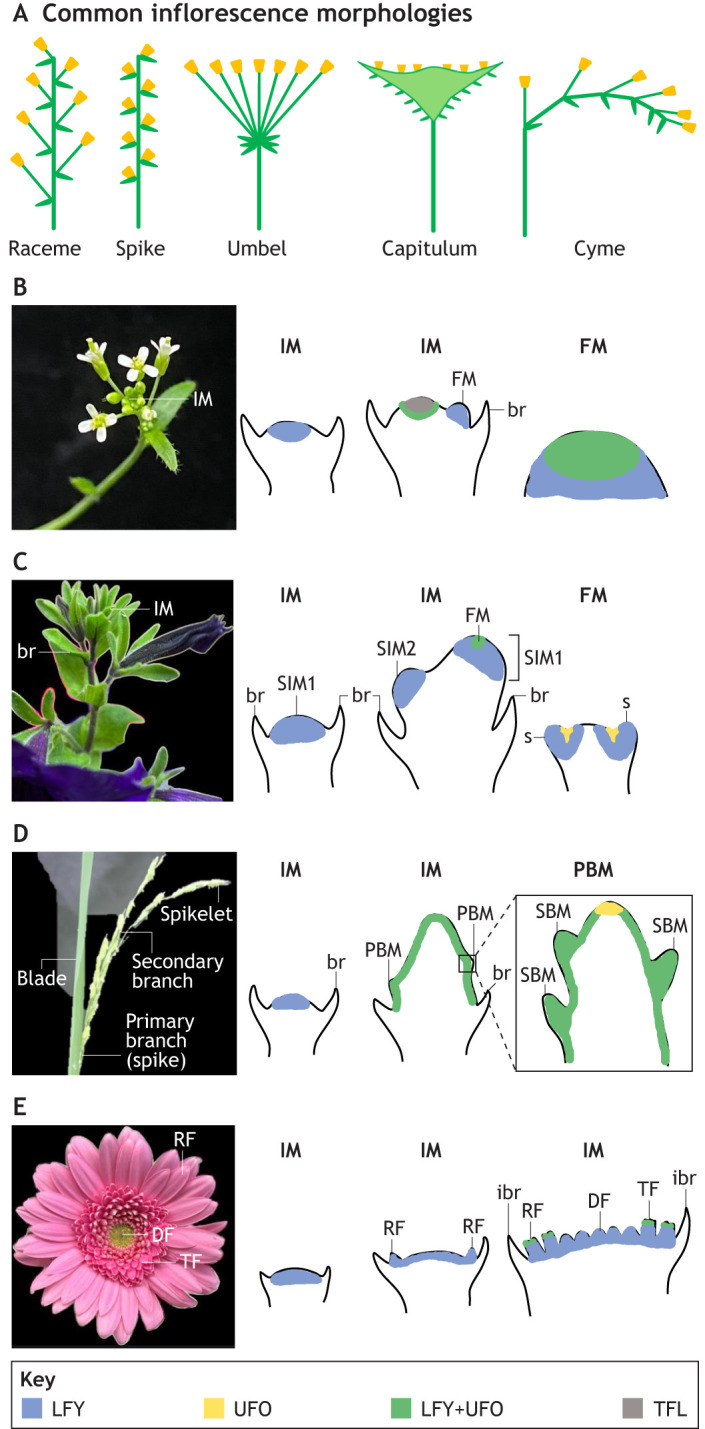
**Inflorescence architectures and schematic representations of *LFY* and *UFO* expression patterns in different inflorescence types.** (A) Schematics of common inflorescence morphologies observed in angiosperms. (B) The raceme of *Arabidopsis thaliana*. The apical meristem is an indeterminate inflorescence meristem (IM) with determinate floral meristems (FM) arising in the axils of the bracts (br). (C) The cyme of *Petunia hybrida*. The sympodial meristem (SIM1) is converted into an FM, and the uppermost lateral meristem takes over as a sympodial meristem (SIM2). The SIM2 is in turn converted to an FM with its uppermost lateral meristem taking over as the next SIM. s, sepal. (D) The panicle of *Oryza sativa*. The IM is indeterminate, forming primary branch meristems (PBMs) on its flanks, which themselves form secondary branch meristems (SBM; shown in the right-hand inset). (E) The capitulum of *Gerbera hybrida*. The IM forms multiple FMs on a flattened head-like structure. Outer FMs may form bilaterally symmetrical ray florets (RF) or smaller ray florets (i.e. trans florets, TF), and inner FMs form radially symmetrical disc florets (DF). The whole structure is subtended by a ring of bracts, known as involucral bracts (ibr). Blue regions correspond to *LFY* expression, yellow regions correspond to *UFO* expression, green regions correspond to *LFY+UFO* expression and grey regions correspond to *TFL* expression. All images taken by the authors.

Inflorescence (see Glossary, Box 1) architecture arises from the first pathway that is initiated in the transition from vegetative to reproductive growth (Fig. 1A,B). A combination of endogenous and exogenous signals converge to convert the vegetative shoot apical meristem (SAM) into an inflorescence meristem (IM) that initiates determinate (see Glossary, Box 1) flower meristems (FMs). The IM is a meristem of the plant that is in the reproductive phase – it usually produces bracts (see Glossary, Box 1), floral meristems and more stem. The FM is the meristem which produces the organs of a single flower. In *Arabidopsis*, the FMs arise in the axils (see Glossary, Box 1) of bracts, as axillary meristems. As a result, the inflorescence is a simple, indeterminate structure that can potentially initiate new flowers indefinitely (Irish, 2010). Acquisition of FM identity by axillary meristems of the inflorescence is controlled by interactions between key positive and negative regulators (Coen and Nugent, 1994). Although inflorescence architecture is regulated by a large GRN in *Arabidopsis*, we concentrate on the core network of *LEAFY* (*LFY*), *APETALA1* (*AP1*), *TERMINAL FLOWER1* (*TFL1*) and *UNUSUAL FLORAL ORGANS* (*UFO*) (Fig. 1B). *LFY* is expressed during the vegetative phase in lateral meristems at steadily increasing levels until it reaches a threshold that directly activates *AP1* to trigger flowering (Weigel et al., 1992; Parcy, 2005; Benlloch et al., 2007). LFY interacts with UFO and activates the expression of multiple floral homeotic genes (Chae et al., 2008). By contrast, *LFY* is repressed in the apical IM by TFL1 (Bradley et al., 1997) (Fig. 2B). Constitutive *LFY* expression converts both the apical meristem (which should ordinarily remain indeterminate and produce further inflorescence) and the axillary meristems (which should either be determinate and form flowers, or remain indeterminate to form inflorescence branches) into terminal flowers (see Glossary, Box 1), indicating that transcriptional regulation of *LFY* is a limiting factor defining spatio-temporal flower development (Weigel and Nilsson, 1995).

### Organ identity

Flowers are composite structures composed of concentric rings of organs called flower whorls (see Glossary, Box 1). Once flowering is initiated, multiple whorls of flower organs arise on each individual FM (Fig. 1A). Patterns of organ initiation vary between species and have a complicated evolutionary history. A perfect flower (see Glossary, Box 1), as exemplified by *Arabidopsis* and *Antirrhinum*, typically contains two outer whorls of sterile organs, and two inner whorls consisting of male (stamens) and female (carpel) reproductive structures. In *Arabidopsis,* four main flower whorls are observed: the sepals (or calyx), petals (or corolla), stamens (or androecium) and termination of floral meristem with the carpel (or gynoecium) (Smyth et al., 1990) (Fig. 1A). Identification of flower homeotic mutants in the late 1980s (Bowman et al., 1989) allowed the formulation of the ABC model for flower development, which defined three classes of genes controlling organ identity: A for sepals, a combination of A+B for petals, B+C for the stamens, and C for the carpel (Bowman et al., 1991; Coen and Meyerowitz, 1991) (Fig. 1C). Later work discovered that protein-protein interactions and formation of protein complexes, mediated by another class of proteins known as the E-class, are important for ABC protein function and control of organ identity (Fig. 1C; Honma and Goto, 2001; Pelaz et al., 2000).

### Flower symmetry

Genetic mechanisms underpinning floral symmetry were first discovered in *Antirrhinum majus*, which displays zygomorphic (i.e. bilaterally symmetrical; see Glossary, Box 1) flowers with two dorsal petals, two lateral petals and one ventral petal, which create a single plane of symmetry with two mirror images (Fig. 1A; Luo et al., 1996). Zygomorphy also extends into the stamens, where the dorsal stamen is aborted after initiation, and a pair of lateral stamens form that are shorter than the ventral pair (Luo et al., 1996). In a radially symmetric *Antirrhinum* mutant, all floral organs in the petal and stamen whorls adopt the ventral identity, creating multiple planes of symmetry. This radially symmetric form results from mutations in two transcription factors: *CYCLOIDEA* (*CYC*) and *DICHOTOMA* (Luo et al., 1996) (Fig. 1D). These two genes were later discovered to be paralogues of the CYC2 clade, belonging to the TCP transcription factor family [named for *TEOSINTE BRANCHED 1* from *Arabidopsis*, *CYC* from *Antirrhinum* and *PROLIFERATING CELL FACTOR* (*PCF*) from maize] (Howarth and Donoghue, 2006). Loss of function of CYC2 genes in *Antirrhinum* results in loss of dorsal identity in petals and stamens, producing a ventralised flower. Ventral identity is controlled by a MYB family transcription factor, *DIVARICATA* (*DIV*), which is expressed throughout petals and stamens of a wild-type flower (Almeida et al., 1997). RADIALIS, an MYB protein directly regulated by CYC2 proteins and therefore localised dorsally within the flower, competes with DIV for DRIF (DIV- and RAD- interacting factor) proteins, inhibiting DIV function in a dosage-dependent manner (Fig. 1D; Corley et al., 2005; Costa et al., 2005; Davies et al., 2006; Raimundo et al., 2013). In the dorsal region, this inhibition is strongest, leading to the dorsal identity, while in the lateral regions the interaction is less strong, leading to the hybrid identity of the lateral petals (Corley et al., 2005; Costa et al., 2005; Raimundo et al., 2013).

### Flower colour

One of the last traits to appear during development is colouration of the flower, which often starts midway through development and continues to mature until anthesis (see Glossary, Box 1) (Fig. 1A). Pigmentation is most often observed in the showy perianth (see Glossary, Box 1) and is best modelled in the colourful *Antirrhinum* flowers, which exhibit a red-pink colour in all petals and a bright yellow region in the three ventralised petals, creating a bicolour pattern. The red pigment is a magenta anthocyanin (Gilbert, 1971) and the yellow pigment is a yellow aurone (Nakayama, 2002). Both pigments are derivatives of the flavonoid biosynthetic pathway (Koes et al., 2005). Regulation of the pathway was first identified in *Petunia* and maize (Paz-Ares et al., 1987; Quattrocchio et al., 1999) and later elucidated in *Antirrhinum* (Schwinn et al., 2006). A trimeric protein complex (called the MBW complex), comprising an MYB family transcription factor, a basic helix loop helix (bHLH) factor and a WD Repeat (WDR) protein, differentially regulates late flavonoid biosynthetic genes (LBGs) in different parts of the flower (Ramsay and Glover, 2005; Rausher, 2008). In *Antirrhinum*, three R2R3 MYB transcription factors – ROSEA1, ROSEA2, and VENOSA – regulate patterning of the red pigment in the petals, with the ROSEA proteins acting widely, while the VENOSA protein acts specifically in the veins of dorsal petals (Fig. 1E) (Schwinn et al., 2006; Shang et al., 2011). A fourth MYB transcription factor, ELUTA (EL), represses anthocyanin production in the petal lobes, creating a bullseye around the opening of the corolla tube (a tube formed from fused petals) (Moss et al., 2024). Two bHLH proteins (DELILA and INCOLORATA I) act within MBW complexes to induce expression of LBGs in the red pigmented regions (Fig. 1E; Martin et al., 1991; Albert et al., 2021). Meanwhile, the WDR protein has not been shown to directly regulate LBGs, but likely acts as a scaffold to aid MBW complex formation (Ramsay and Glover, 2005; Jain and Pandey, 2018). In addition to these regulators of red anthocyanin, a silencing RNA found in the *SULFUREA* (*SULF*) locus leads to a spot of yellow pigment being localised to the opening of the corolla tube (Whibley et al., 2006; Bradley et al., 2017).

The four GRNs discussed above ensure the production of a functional flower, from initiation to pigmentation (Fig. 1). These four modules are themselves subject to alteration by or interaction with key developmental mechanisms including boundary formation (Rebocho et al., 2017), tissue polarity (Bowman and Floyd, 2008; Green et al., 2010), heterochrony (Coen and Prusinkiewicz, 2024) and mechanical forces (Abad et al., 2017), and tinkering with any of these developmental mechanisms can also alter the morphology of floral display. However, we focus here on the four modules described because they represent key developmental constraints, which when modified have been associated with diversification and speciation of angiosperms (Theissen and Melzer, 2007; Litt and Kramer, 2010; Yuan et al., 2016). Several hypotheses have suggested that central node genes, those with lowest pleiotropy and highest phenotypic effect, are often direct targets of selection and affect the evolvability of a trait (Gompel and Prud‘homme, 2009; Martin and Orgogozo, 2013; Streisfeld and Rausher, 2011; Pavličev and Cheverud, 2015). Therefore, although each of the major GRNs involves multiple components and complex interactions with small RNAs, proteins and chromosome architecture, we focus here on the key central genes likely associated with the evolvability of the corresponding trait.

## Complex floral displays

Biological complexity has historically been defined across organisational scales (Adami et al., 2000; Wolf et al., 2018). Often, complexity is referred to at the level of the genome (genome size, chromosome number, structure of genes; Ren et al., 2018), cells (types, density, interactions, size; Wolkenhauer and Muir, 2011) or tissues (cell types, interactions, layer organisations; López-Martínez et al., 2024). Attempts to study and explain morphological complexity of angiosperms have tried to reduce descriptions of morphology to simple codes (Burleigh et al., 2006), to characterise reproductive structures as modular organs (Leslie and Mander, 2023) and to confine complexity to functional outputs for pollinators (Krishna and Keasar, 2018). These definitions often omit development as an integral part of complexity. In this Review, we define complexity in floral display as being produced by developmental variations among four structural traits common to all angiosperms and arising from the modules discussed above: (1) inflorescence architecture, (2) organ identity, (3) flower symmetry and (4) flower colour (Fig. 1). We define intermediate complexity as variation within one of these traits due to developmental changes, which give rise to new and intricate phenotypes that differ from the canonical, simple models (Box 2A-D). Examples of intermediate complexity for the purposes of this Review could therefore include variation of flower colour such as spots and stripes, which often consist of integration and co-option of disparate biosynthetic and gene regulatory pathways (Box 2D). Meanwhile, we consider high complexity to involve changes in more than one of the four primary characteristics. These changes could include variation in two modules, such as inflorescence architecture and organ number, inflorescence architecture and floral symmetry, inflorescence architecture and floral colour, and so forth (Box 2E-J). Alternatively, they could include variation in more than two of these primary characteristics (Box 2K-P). For example, *Amorphophallus titanum* Becc (Box 2M) exhibits modifications to inflorescence structure, organ identity and floral colour when compared with simple model species, while the daisy *Gorteria diffusa* Thunb. has altered floral organs and symmetry, and complex sexually-deceptive petal spots with spatial tuning (Box 2P). In this section, we discuss how changes to the GRN of each of the four floral display modules has resulted in more complex forms of that module.

**Box 2. Complex floral displays**

In this Review, we describe complexity among four structural traits common to flowering plants: (1) inflorescence architecture, (2) organ identity, (3) floral symmetry and (4) floral colour. (A-D) We define intermediate complexity as variation from the model system within one of these traits because of developmental changes, which gives rise to new and intricate phenotypes such as those shown here: (A) *Syringa vulgaris* with complex inflorescence [image courtesy of Sally Petitt and Cambridge University Botanic Garden (CUBG)]; (B) *Dietes robinsoniana* with modified perianth organ identity; (C) *Pholidota imbricata* with orchid zygomorphy; (D) *Hibiscus trionum* with bullseye colour patterning. (E-P) High complexity results from changes in two or more of the four primary characteristics: (E) *Asclepias syriaca* with complex inflorescence and modified floral organs (image courtesy of Sally Petitt and CUBG); (F) *Myosotis arvenis* with cymose inflorescence and bicolour corolla; (G) *Anthriscus sylvestris* with complex inflorescence and both zygomorphic and actinomorphic flowers; (H) *Linaria spartea* with zygomorphy determining the position of elaborated nectar spurs (image courtesy of Joaquín Ramiérez, José Quiles, Pablo Vargas through CUBG); (I) *Helleborus orientalis* with modified floral bracts and colour patterning; (J) *Petrea volubilis* with petaloid sepals and zygomorphic stamens; (K) *Euphorbia rigida* with complex inflorescence, modified floral organs and zygomorphy (image courtesy of Sally Petitt and CUBG); (L) *Veronica gentianoides* with modified floral organs, zygomorphy and colour patterning; (M) *Amorphophallus titanum* with complex inflorescence, modified organ identity and colour patterning (image courtesy of Howard Rice and CUBG); (N) *Leucadendron discolor* with complex inflorescence, modified organ identity and colour patterning; (O) *Passiflora caerulea* showing modified floral organs, colour patterning and variation of symmetry between whorls; (P) *Gorteria diffusa* (Buffels) Thunb. showing a daisy capitulum which affects floral organs and symmetry, and complex sexually-deceptive petal spots. Photographs were taken by the authors unless specified otherwise.
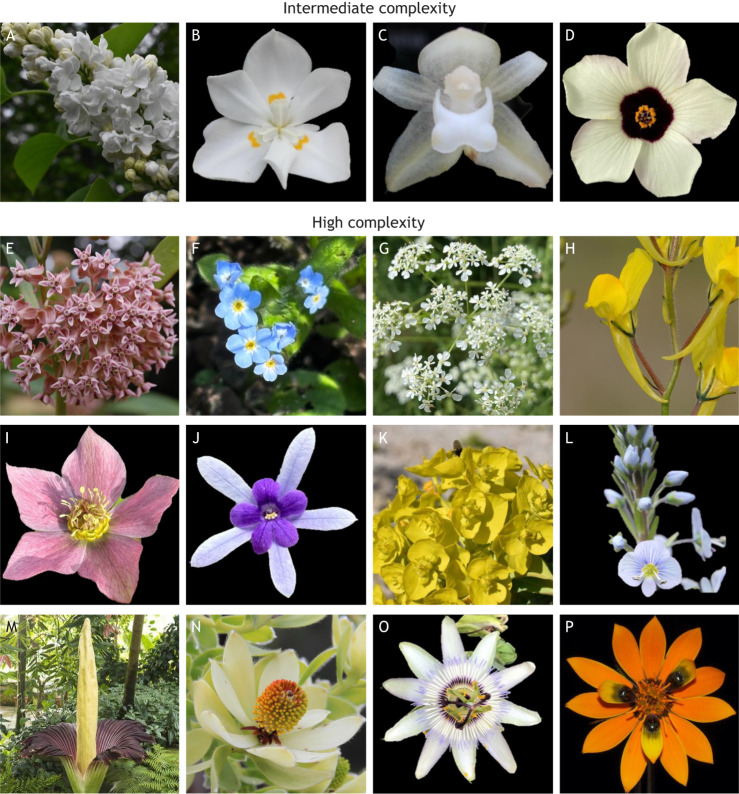

### Inflorescence architecture

In an indeterminate inflorescence, the apex does not lose its meristematic identity and continues to produce flowers from lateral positions indefinitely, or to produce further IMs from lateral positions to produce branched, compound inflorescences. Based on the initiation pattern of lateral meristems around the stem and the patterns of internode lengths, an indeterminate inflorescence can be classified as a raceme (in the case of simple flowers such as *Arabidopsis*, *Antirrhinum* and *Linaria*), or as one of a number of more complex forms including a panicle (e.g. rice and wheat), umbel (e.g. carrot) or capitulum (e.g. *Gerbera* and sunflower) (Fig. 2A; Weberling, 1989; Coen and Nugent, 1994; Benlloch et al., 2007). In contrast, in a determinate inflorescence, the SAM converts to an FM after flowering initiation, which terminates with differentiation of the carpels. The inflorescence may therefore consist of a solitary flower or, more commonly, produce a new apex from the uppermost lateral meristem. Repeated termination of the apical meristem with development of new branches creates a cyme (e.g. *Petunia*) (Fig. 2A) (Souer et al., 2008). Here, we discuss how three complex inflorescence architectures (panicle, capitulum and cyme) result from key changes to and fine tuning of LFY and UFO activity. We do not discuss umbel formation here, as the mechanisms underpinning this inflorescence architecture are not currently well defined.

The inflorescence of *Petunia hybrida* Regel (Solanaceae) is a typical cyme in which the apical meristem terminates by forming a flower and growth continues from a lateral meristem (Fig. 2C). In the IM, expression of *ABERRANT LEAF AND FLOWER* (*ALF*), the *Petunia* orthologue of *LFY*, appears first in the apical region and, with a slight delay, in the emerging lateral meristem that forms the next sympodial inflorescence meristem (SIM) (Souer et al., 2008). In *Petunia*, ALF activity is spatially and temporally restricted through *DOUBLE TOP* (*DOT*), a petunia orthologue of *UFO*. Early expression of *DOT* is found in the apical FM, while expression in the uppermost lateral meristem is delayed, appearing after the expression of *ALF* (Souer et al., 2008). Transgenic overexpression of *DOT* across the inflorescence results in conversion of the whole cyme into a single flower (Souer et al., 1998, 2008; Moyroud et al., 2010). This contrasts to the situation in *Arabidopsis*, where *UFO* transcripts are detectable at the apices of all meristems, but the vegetative SAM and the IM are not converted to flowering by UFO because there is no equivalent expression of *LFY* (Fig. 2B)*.* This absence of *LFY* transcript also prevents ectopic expression of *UFO* from converting the IM to an FM in *Arabidopsis* (Weigel and Nilsson, 1995; Lee et al., 1997; Moyroud et al., 2010).

The rice (*Oryza sativa* L., Poaceae) panicle inflorescence is complex because it contains both primary (spike) and secondary (spikelets) branched inflorescence structures, together forming a panicle (a much-branched inflorescence) (Fig. 2D). This compound inflorescence requires the initiation and maintenance of a series of indeterminate meristems from which the determinate floral meristems arise. The initial transition to the inflorescence state is specified by *RICE FLORICAULA LEAFY* (*RFL*), an *LFY* orthologue. *RFL* is expressed early in panicle IM development and, like *Arabidopsis LFY*, is necessary for development of all reproductive organs, including flowers (Fig. 2D) (Kyozuka et al., 1998). However, unlike *LFY* in *Arabidopsis*, *RFL* expression is retained throughout the IM, and it acts to maintain the IM in the indeterminate state (Kyozuka et al., 1998; Rao et al., 2008). *RFL* expression is dynamic in the subsequent branch meristems [both primary (PBMs) and secondary (SBMs)], where it is high at some stages but transient, a pattern that appears to be important in maintaining inflorescence architecture (Rao et al., 2008). *ABERRANT PANICLE ORGANIZATION1* (*APO1*) is the rice orthologue of *UFO*, which works with *RFL* in the maintenance of IM indeterminacy (Ikeda et al., 2007). *RFL* also controls branching at the whole plant level, as silencing of *RFL* abolishes the development of tillers (secondary shoots growing from the base of the rice plant) (Kyozuka et al., 1998; Ikeda et al., 2007; Rao et al., 2008; Ikeda et al., 2019; Moyroud et al., 2010). Studies in which *RFL* expression is downregulated or *APO1* is mutated have shown that both genes are necessary to maintain branch number within the inflorescence, and that loss of their activity results in a determinate IM, which terminates in a spikelet (Kyozuka et al., 1998; Ikeda et al., 2007; Rao et al., 2008; Ikeda-Kawakatsu et al., 2009). This suggests that regulation of this key module and its expression domain is crucial for defining the number of inflorescence structures arising in rice and therefore the overall form of this complex branched inflorescence.

*Gerbera hybrida* Hort. belongs to the Asteraceae family and produces a capitulum (Fig. 2E; Zhang and Elomaa, 2024). The capitulum is a condensed inflorescence, resulting from loss of the internodes, which leads to a single flower mimic. A *Gerbera* capitulum contains an outer ring of ray flowers (or ray florets; strongly bilaterally symmetrical flowers that superficially resemble single enlarged petals), a ring of smaller ray flowers (or trans florets) and central spirals of disc flowers (or disc florets; small, radially symmetrical flowers that together form the central ‘disc’ of the daisy structure). *Gerbera GhLFY* is uniformly expressed across the entire IM, which means that the capitulum is determinate, more closely resembling a single FM than a conventional indeterminate IM (Zhao et al., 2016) (Fig. 2E). Suppression of *GhLFY* expression in transgenic RNAi lines leads to loss of floral organ identity and conversion of the ray floret primordia into branched peripheral units, meaning that the capitulum no longer closely resembles a single flower (Zhao et al., 2016). Moreover, the *GhLFY* expression domain is associated with early ontogeny of ray florets emerging in the axils of the involucral bracts (the leaf like structures subtending the inflorescence) (Zhao et al., 2016). In contrast, *GhUFO* is not expressed in the IM but in the emerging floral primordia, where it plays the same role in regulating FM identity as its orthologue in *Arabidopsis* (Zhao et al., 2016). Expression of *GhUFO* across the IM using a constitutive promoter causes the capitulum to develop as a single flower with many whorls of organ primordia (Zhao et al., 2016; Elomaa et al., 2018). In the case of *Gerbera*, then, the roles of *LFY* and *UFO* have become specialised, such that expression of *LFY* across the whole IM reflects the functional behaviour of the capitulum as a single flower, whereas expression of *UFO* only in the FMs ensures that the capitulum only produces florets, not meristems capable of more extensive or branched growth.

These examples illustrate how changes in the expression patterns of key transcriptional regulators have allowed for enormous morphological diversity of inflorescence architectures. However, it is likely that changes in protein function are also involved. Recent work has demonstrated that direct protein-protein interaction between LFY and UFO in *Arabidopsis* is necessary to activate the expression of target genes that lack clear LFY binding sites in their promoters (Rieu et al., 2023). It is very likely that differences in the interdependency between LFY and UFO in different species, and the consequent effects on inflorescence architecture, can be attributed to changes in the biochemical interactions between the proteins and between the proteins and the DNA targets that they bind.

### Organ identity

As discussed above, the identity of all flower organs is specified during early stages of flower development by a subset of the MADS-box family of transcription factors, known as the ABCE genes (Fig. 1C; Bowman et al., 1991; Coen and Meyerowitz, 1991; Theissen and Gramzow, 2016). Here, we discuss how changes to organ identity and the evolution of novel organ types and organ outgrowths, when combined, lead to the enormous phenotypic diversity of flowering plants.

**Fig. 3.**
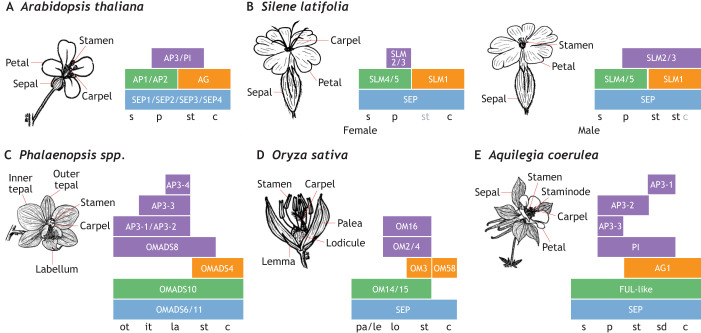
**Variation in flower organ number and identity is related to floral display complexity.** (A-E) Schematics of the ABC model that underpins the formation of floral organs in selected species, showing the final floral architecture and the underlying genes contributing to organ identity. The identity of floral organs is controlled by combinatorial action of the ABC genes. Colours of the boxes indicate the class of genes (A, green; B, purple; C, orange; E, blue). The organ identities defined by these gene combinations are shown at the bottom of the schematics (c, carpel; it, inner tepal; la, labellum; le, lemma; lo, lodicule; ot, outer tepal; p, petal; pa, palea; s, sepal; sd, staminode; st, stamen). (A) The canonical ABC model was first described in *Arabidopsis.* Different combinations of A, B, C and E gene expression promote the formation of sepals, petals, stamens and carpels. For example, regions where the B, C and E class genes are expressed form stamens, and regions where the A and E class genes are expressed form sepals. AG, AGAMOUS; AP1/2/3, APETALA 1/2/3; PI, PISTILLATA; SEP1/2/3/4, SEPALLATA 1/2/3/4. (B) In female *Silene latifolia* flowers (left), repression of B function expression in whorl 3 underpins the loss of stamens. In male *S. latifolia* flowers (right), extension of B function expression into whorl 4 explains the loss of carpels*.* SLM1/2/3/4/5, SILENE LATIFOLIA MADS 1/2/3/4/5; SEP, SEPALLATA. (C) Extensive duplication within B class genes is thought to explain the diversity of tepal morphology in orchids, which form inner and outer tepals, such as those of *Phalaenopsis spp*. AP3-1/3-2/3-3/3-4, APETALA3-1/3-2/3-3/3-4; OMADS4/6/8/10/11, ORCHID MADS 4/6/8/10/11. (D) Rice florets exhibit ABC model expression patterns that are very similar to the canonical ones, but changes in the targets of A and B function genes lead to the novel morphologies of the lemma, palea and lodicule. OM2/3/4/14/15/16/58, ORYZA SATIVA MADS 2/3/4/14/15/16/58; SEP, SEPALLATA. (E) In *Aquilegia* flowers, the duplication and diversification of the B function genes enables the development of a staminode (sterile stamens) whorl in addition to the usual four whorls. AG1, AGAMOUS 1; AP3-1/3-2/3-3, APETALA 3-1/3-2/3-3; FUL-like, FRUITFUL-like; PI, PISTILLATA; SEP, SEPALLATA.

Changes to organ identity have contributed to phenotypic diversity in angiosperms. Most flowers, including *Arabidopsis* (Fig. 3A) are perfect (i.e. hermaphroditic), housing both male and female organs (Renner, 2014). However, dioecy – separation of male and female reproductive organs on flowers of different individuals – has evolved across multiple clades and is often considered an evolutionary dead-end, as it prevents self-fertilisation and limits opportunity to expand the plant's distribution range (Käfer et al., 2017). Variations between perfect and dioecious flowers include monoecy, andromonoecy, gynomonoecy, androdioecy and gynodioecy (see Glossary, Box 1; Renner, 2014). Suppression of either male or female organs often requires a complicated GRN, predicted as either linkage of sex-determining genes on a single chromosome (e.g. the sex-determining chromosome) or co-regulation of multiple genes, including MADS-box genes (Charlesworth, 2016). In dioecious *Silene latifolia* Britten & Rendle, where individual plants have either male only or female only flowers, changes in expression of *SLM2* and *SLM3*, orthologues of B-class genes from *Arabidopsis*, are strongly associated with carpel development in female flowers and carpel repression in male flowers (Fig. 3B; Hardenack et al., 1994; Bačovský et al., 2021 preprint). These changes in organ number and identity lead to changes in not only the functional biology of the plant, but complex floral displays.

In bisexual flowers, reproductive organs are usually conserved while sterile organs (i.e. the perianth) are more labile to evolutionary selective pressures (Takhtajan, 1991). The perianth is thought to have evolved from subtending leaves of reproductive structures, although in several lineages petals evolved independently from stamen modifications (Takhtajan, 1991). In monocots, the perianth is commonly (although not always) undifferentiated (meaning that the sepals and petals do not look different) so these organs are collectively referred to as tepals (divided into inner and outer tepals; see Glossary, Box 1), while in eudicots the perianth is more usually differentiated into sepals and petals (Remizowa et al., 2010). Petaloid appearance of a perianth organ is often associated with B-class function across diverse angiosperm lineages (Kramer and Jaramillo, 2005). Within orchids, tepals are highly elaborate, with distinct inner and outer tepals, which may in part be a result of the activities of a higher number of B-class genes (Fig. 3C; Su et al., 2013). Distinct expression patterns of orchid B class genes and novel protein-protein interactions of their protein products have been integrated into a model which postulates that competition between protein complexes for different B-class proteins controls the formation of the distinct and elaborate inner and outer perianth organs in orchids (Hsu et al., 2015; Teo et al., 2019). The petaloid bracts of *Cornus* (Feng et al., 2012) and *Davidia* (Vekemans et al., 2012) result from extended B-class function into the subtending bracts. Similarly, B and E class function have expanded to produce the petaloid sepals of *Impatiens* and *Marcgravia* (Geuten et al., 2006). These cases illustrate how duplications and subfunctionalisation of B-class genes are associated with diverse petal morphologies and identities in angiosperms.

The evolution of novel floral organs and organ outgrowths have also contributed to the diversification of angiosperms. Novel organs include staminodes (in *Aquilegia*; see Glossary, Box 1), pappus (in Asteraceae) and grass perianth organs, while organ outgrowths include nectar spurs (in *Linaria*) and the corona (in *Narcissus*). In grasses, the perianth is differentiated into lemma, palea and lodicules (Fig. 3D). The lodicules are considered homologous to petals, while the lemma and palea are often considered homologous to sepals (Yoshida and Nagato, 2011). The identity of these novel organs is also determined by the action of A- and B-class genes. In the case of rice (Fig. 3D), *OsMADS14* and *OsMADS15* belong to the A-class, whereas *OsMADS2*, *OsMASDS4* and *OsMADS16* are B-class genes (Zhu et al., 2022). These MADS-box proteins display similar protein interaction behaviours to ABCE MADS proteins from model species such as *Arabidopsis* (Fig. 3A), suggesting that it is the downstream targets that have changed to allow morphological evolution (Kater et al., 2006; Dreni and Kater, 2014). In *Aquilegia*, two novel morphologies are observed: nectar spurs (organ outgrowths) (Kramer, 2009) and staminodes (Fig. 3E; Sharma and Kramer, 2013). *Aquilegia* spurs develop as a bulge of cells, resulting from cell proliferation, at the base of the petal primordium, followed by anisotropic cell elongation (Puzey et al., 2012). Recent studies indicate that spur development is controlled by multiple factors (Edwards et al., 2022), including brassinosteroids (Conway et al., 2021) and several transcription factors such as *AqPOPOVICH* (Ballerini et al., 2020) and *AqTCP4* (Yant et al., 2015). Staminodes, however, are sterile stamens, which usually display differences in morphology from fertile stamens such as a petal-like lateral expansion of the filament or anther tissue (Ronse de Craene and Smets, 2001; Appleton and Kramer, 2024). In *Aquilegia*, staminodes have evolved from sub- and neofunctionalisation of *APETALA3* paralogues (Sharma and Kramer, 2013).

These cases illustrate co-option of diverse genetic and chemical pathways in generating novel floral organs in addition to modifications of the downstream targets of ABCE genes. Together, modifications in organ identity and number allow for diverse and complex floral displays.

### Floral symmetry

**Fig. 4.**
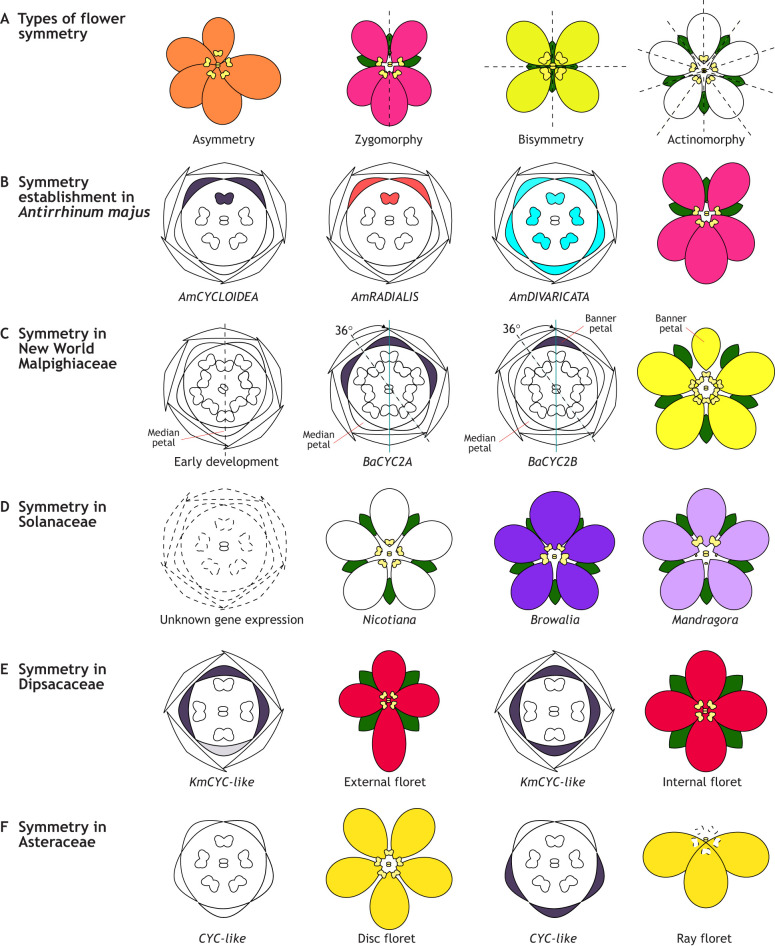
**Schematics of diverse flower symmetries and their developmental patterns.** (A) Schematics showing different types of flower symmetry patterns, where dashed lines represent the axes of symmetry. (B-F) Schematics illustrating how flower symmetry develops in different species. Flower diagrams show sepals, petals, stamens and carpels from the outside-in. Gene expression patterns are superimposed on the diagrams of the developing flowers for which expression data are available; black represents *CYC-*like gene expression, red represents *RADIALIS* gene expression and blue represents *DIVARICATA* gene expression. (B) During flower symmetry development of *Antirrhinum majus*, CYC2 genes and their target *RAD* are spatially restricted to the dorsal region of the flower, inhibiting *DIV* activity in the dorsal region and leading to dorsal petal identity and an aborted dorsal stamen. (C) In New World Malpighiaceae zygomorphic flowers, there is a 36-degree rotation from the incipient axis of symmetry (dashed line) to the final axis of symmetry (solid line) during development, which is likely regulated by the dorsal restriction of CYC2 genes, and particularly the expression of *BaCYC2B* in one of the two dorsal petals. This leads to the formation of the banner petal and the rotation of the axis of symmetry. (D) Schematics show Solanaceae floral phyllotaxis and symmetry patterns, including actinomorphic *Nicotiana* (white), zygomorphic *Browallia* (dark purple) and the actinomorphic corolla with zygomorphic stamens of *Mandragora* (light purple). Little is known about the underlying mechanisms leading to these alternate symmetry patterns between organ whorls of Solanaceae flowers. (E) Expression of *CYC*-like genes superimposed on floral diagrams of external and internal florets of *Knautia* (Dipsacaceae). Differences in the expression of *CYC*-like genes in the ventral region lead to differences in morphology between the florets. (F) Expression of *CYC*-like genes superimposed on floral diagrams of disc and ray florets of Asteraceae. In actinomorphic disk florets, the expression of the *CYC*-like genes is the same (either present or absent) across all petals, whereas in zygomorphic ray florets it is differential between dorsal and ventral petals.

Arrangement of floral organs within the flower may generate multiple plane(s) of symmetry which, when drawn through the face of the flower, generate exact mirror images. Thus, flowers can be classified as asymmetric (no planes of symmetry), zygomorphic (i.e. bilateral, monosymmetric; one plane of symmetry), bisymmetric (two planes of symmetry) or actinomorphic (i.e. radial, polysymmetric; multiple planes of symmetry; Fig. 4A; see Glossary, Box 1). Actinomorphy is often considered the ancestral and default state of flowers (Reyes et al., 2016; Sauquet et al., 2017). However, floral symmetry can vary among different flower whorls, and among flowers on an inflorescence stalk. Variations in floral symmetry include aestivation (contortion of the flower to the left or right), chirality (spiral rotation of organ attachment) and presentational enantiostyly (angle of presentation of the female reproductive structure). Recent studies estimate that there have been at least 199 transitions in floral symmetry in angiosperms, most occurring from actinomorphy to zygomorphy (Reyes et al., 2016). However, despite its prevalence in angiosperms, zygomorphy occurs in many forms, and three different symmetry patterns (defined by the ratio of dorsal to ventral petals: 2:3, 4:1 or 0:5) are found within Asteridae alone (Donoghue et al., 1998). Here, we discuss how complex symmetry patterns occur through: (1) shifts in symmetry during development, (2) variation in symmetry between floral whorls and (3) variation of symmetry within an inflorescence.

Initial cues for floral symmetry are often physically initiated by subtending bracts and/or bracteoles (Endress, 2010), but later demarcated by symmetry GRN, as illustrated in *Antirrhinum* (Figs 1D and 4B). Recently, nine primary developmental patterns of zygomorphy establishment were identified among core eudicots (Bukhari et al., 2017). During floral organ initiation in core eudicots, petals are arranged with an incipient symmetry respective to the stem, where a single petal (the median petal) is either positioned in the abaxial (away from the main growth axis) or adaxial (towards the main growth axis) position. Median-abaxial (MAB) petal initiation is the most common and plesiomorphic condition among core eudicots, while median-adaxial (MAD) petal initiation evolved at least 28 times (Bukhari et al., 2017). During symmetry specification, the axis of symmetry can rotate relative to the median plane of the flower. These rotations occur along 0, 36, 72, 108, 144 and 180° angles for the MAB pattern, and along 0, 72 and 108° angles for the MAD pattern (Bukhari et al., 2017). MAB petal initiation was shown to be ancestral to the Malpighiaceae family, followed by a 36° rotation leading to a mature flower with a central dorsal petal display (Zhang et al., 2010; Bukhari et al., 2017). This shift in symmetry results from changes in the expression pattern of CYC2 genes in the Malpighiaceae, the genomes of which contain two CYC2 paralogues, predating the diversification of the family (Zhang et al., 2010, 2012, 2013, 2016). A shift in expression of one of the paralogues from the two dorsally positioned petals to only one of them rotates the angle of symmetry by 36° (Fig. 4C; Zhang et al., 2010, 2012, 2013, 2016). Thus, lability of flower symmetry across development is directly regulated by differential expression of key transcription factors and leads to evolution of complex flower symmetry forms.

Often, zygomorphy is only observed in the petals, or petals and stamens. However, symmetry changes may affect all four flower whorls and in different ways. One of the best examples of different symmetry patterns amongst floral whorls is observed in Solanaceae, which contains species with fully actinomorphic flowers (*Nicotiana obtusifolia* Martens & Galeotti) and fully zygomorphic flowers (*Browallia speciosa* Hook.), as well as flowers with zygomorphic stamens but actinomorphic petals (*Mandragora caulescens* Clarke) (Fig. 4D; Bohs et al., 2007; Knapp, 2002). Recently, Zhang et al. (2017a,b) reinforced the proposition that changes in symmetry of stamens and petals of Solanaceae evolved separately but are correlated, with zygomorphy in the stamens being more common and having likely preceded zygomorphy in the petals (Knapp, 2010; Robyns, 1931). The working hypothesis is therefore that differential regulation of symmetry operates between the different whorls, but this requires further investigation to unravel the specific molecular mechanisms involved in these diverse floral symmetry patterns of Solanaceae.

Flowers occurring on the same inflorescence may also have drastically different symmetry patterns. In species with a simple inflorescence structure, like the raceme of *Antirrhinum*, flowers appear regularly with the same symmetry pattern, although the terminal flower may occasionally be actinomorphic when all others are zygomorphic (Bradley et al., 1996). In condensed capitulum inflorescences, such as those of Apiales, Asteraceae and Dipsacales, the outermost flowers are often zygomorphic, while the innermost flowers are actinomorphic (Donoghue et al., 1998). Study of the genetic underpinnings of symmetry development has revealed that gene duplications followed by subfunctionalisation are associated with contrasting symmetry patterns within a single inflorescence (Howarth and Donoghue, 2006). In Dipsacales, duplications of *CYC*-like genes are observed across all three known paralogues (i.e. *CYC1*, *CYC2* and *CYC3*), resulting in six *CYC*-like genes (Carlson et al., 2011). In *Knautia*, five of these six *CYC* paralogues are differentially expressed in the single dorsal and two lateral petals of the external flowers, while in the internal flowers *CYC* expression is expanded to the ventral region, thus expanding the region of dorsal identity throughout the flower (Fig. 4E; Berger et al., 2016). Similarly, in the Asteraceae, duplications of CYC2 genes followed by subfunctionalisation and restricted expression domains are associated with differences between zygomorphic ray and actinomorphic disc florets on the capitulum of *Senecio*, *Gerbera* and *Helianthus* (Kim et al., 2008; Broholm et al., 2008; Chapman et al., 2012). In *Helianthus annuus* L., at least ten *CYC*-like copies, distributed among three CYC clades, have been isolated (Chapman et al., 2012). Notably, five paralogues within the CYC2 clade show differential expression in the ray florets, where they are associated with zygomorphy, and have low or no expression in disc florets (Chapman et al., 2012). In *Senecio*, two naturally occurring capitulum morphs are found: radiate (with zygomorphic ray and actinomorphic disc florets) and discoid (with only actinomorphic disc florets) (Fig. 4F) (Kim et al., 2008). Overexpression of *SvRAY1*, a homologue of *Helianthus* and *Gerbera CYC2*, from discoid *Senecio vulgaris* leads to repression of ray florets in the radiate background, with either short ligules or no ray florets at all (Kim et al., 2008). This demonstrates the crucial role of CYC2 genes not only in specifying symmetry, but in fact in determining the ray floret identity in Asteraceae. Thus, these examples demonstrate some of the selective pressures acting on CYC loci through gene duplication, and the tissue-specific regulation that is associated with zygomorphy evolution across angiosperms.

### Floral colour

Flower colour is nearly exclusive to the petaloid organs and is one of the primary indicators to biotic pollinators, which are often attracted to bright and contrasting colours (Narbona et al., 2021). In principle, flower colour is dependent on both reflectance of certain wavelengths of light and subsequent perception of colour (Whitney et al., 2009; Kellenberger and Glover, 2023). The former is primarily an effect produced by plant pigments, chemicals which absorb certain wavelengths of light and reflect others, while the latter depends on the ability of the perceiving organism to perceive and interpret colour (e.g. perception of UV light by birds and bees, but not humans). Furthermore, flower colour is incredibly labile and can vary within an individual (e.g. *Hydrangea*), across populations (e.g. *Clarkia*) and between species (e.g. *Mimulus*).

Colour in angiosperms is mainly produced by pigments from two major classes: flavonoids and carotenoids. Both classes are ancient, likely having evolved with light harvesting for photosynthesis, and form precursors to major compounds involved in cell signalling, UV protection and various cofactors involved during development (Koes et al., 2005; Grotewold, 2006; Tanaka et al., 2008; Zhu et al., 2010). A third class, betalains, has evolved exclusively in the Caryophyllales order and represents a further diversification of pigmentation in angiosperms (Timoneda et al., 2019). Restriction and accumulation of pigments within vacuoles or chromoplasts of the petaloid epidermis has likely evolved for pollinator attraction (Koes et al., 2005; Grotewold, 2006; Tanaka et al., 2008; Zhu et al., 2010). Together, these pigments cover the broad range of the visible spectrum of light: white flavones, yellow aurones, red and blue anthocyanins and orange carotenoids. However, pigments can be modified chemically through cofactors of the main organic compound, which alter its chemistry and subsequent colour. Examples include oxygen binding to carotenoids (e.g. *Tagetes erecta*; Thirumalmurugan et al., 2020) or metals binding to anthocyanins (e.g. *Meconopsis grandis*; Yoshida et al., 2006). These organic compounds are further dependent on cellular conditions such as temperature and pH, which can alter their chemistry and absorbed colours. Here, we discuss complexity in flower colour through: (1) structural modifications and (2) patterning of pigments.

One way to generate complex colour patterns is by modifying the structure of the epidermis. Modifications to cell morphology and cuticle composition affect the overall colour of the flower. For example, conical cells, found on the epidermis of most petaloid structures, focus light to underlying vacuoles, causing greater absorption of light that intensifies the hue of the pigment (Noda et al., 1994; Gorton and Vogelmann, 1996). Other notable structures like papillae, trichomes, photonic crystals and overall cuticle composition are starting to be investigated in flowering plants (Thomas et al., 2009; Vignolini et al., 2013, 2014; Moyroud et al., 2017; Wilts et al., 2018; Kellenberger et al., 2023; Fattorini et al., 2024). These epidermal modifications can create multidimensional structures that can interfere, diffract or scatter light, producing complex colours (e.g. iridescence) (Whitney et al., 2009; Sun et al., 2013). Some features may be more ancient than previously thought, such as the blue iridescence in *Selaginella* (Whitney et al., 2009) and the photonic crystals of brown algae (Lopez-Garcia et al., 2018).

**Fig. 5.**
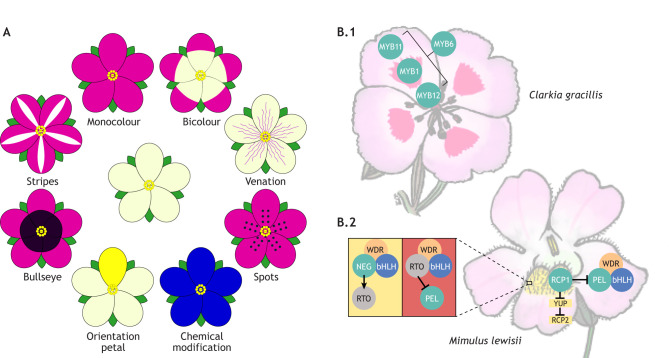
**Diversity of flower colour patterning and developmental patterns.** (A) Schematics of known flower colour patterns. The central flower has unpigmented petals. The top-left schematic shows a monocolour flower, with pink pigmentation in all petals. Moving clockwise, the bicolour flower has pigmented (pink) and unpigmented regions to each petal. Venation patterning is shown next, with pink pigmentation over the veins of the petals. Below that, a flower is shown with pink pigmentation and darker pigmented spots. At bottom-right, the flower is blue because chemical modification has changed the absorption spectrum of the pigment. The bottom-left schematic shows a flower in which only one petal is pigmented yellow, providing an orientation petal to aid pollinator foraging. The next flower has two different pigment colours on each petal, creating a central bullseye pattern. The final flower has pink pigment but an unpigmented region over the midrib of each petal, creating stripes. (B) Regulation of pigment synthesis in *Clarkia gracilis* (B.1) and *Mimulus lewisii* (B.2). *C. gracilis* has four paralogues of R2R3 MYB transcription factors of which three, *MYB6*, *MYB11* and *MYB12*, show expression patterns associated with the background pink pigment production (*MYB11*, distal part of the petal; *MYB12*, proximal part of the petal; *MYB6*, whole petal), while *MYB1* is only associated with the red spot region. Furthermore, *MYB12* exclusively regulates pigment production in the white region at the base of the petals. In *M. lewisii*, PELAN (PEL) forms an MBW complex (with an unknown bHLH and WDR partner) to produce the pink colour in petals, while RCP1 inhibits and restricts PEL in the ventral petal ridges and, through a double negative regulation of YUP and RCP2, produces yellow pigment. Meanwhile, a reaction-diffusion gradient comprised of NEGAN (NEG) and its R3 MYB repressor RTO, produces the red spots on the ventral petal ridges.

Another way to generate complex colours in flowering plants is through patterning and integration of multiple pigment classes and structural elements. Multiple colour patterns are found in angiosperms (Fig. 5A), with the more complex patterning forms associated with visual deception, such as mimicry of another plant's morphology or pollinator (e.g. *Ophrys*; Kellenberger et al., 2023). Biosynthetic pathways producing pigments have been well characterised across flowering plants (Tanaka et al., 2008) and their regulation is modelled in *Antirrhinum* (discussed above and in Fig. 1E). However, evolution of complex colour patterns is often associated with differential expression and regulation of LBGs, as targeting early parts of the pathway is likely to have deleterious and pleiotropic affects (Rausher, 2008). American *Clarkia gracilis* Nelson & Macbr displays a complex colour pattern comprised of a bicolour pattern with a pink background, a red spot, and a rarely observed white cup region (Lin and Rausher, 2021). Position, size and patterning of all three colour elements varies across subspecies of *C. gracilis* (Martins et al., 2017). Four paralogous R2R3 MYB transcription factors in *C. gracilis* likely arose through separate gene duplications (Lin and Rausher, 2021). Three MYBs (*CgMYB6*, *CgMYB11*, *CgMYB12*) show expression patterns associated with the background pink pigment production (Lin and Rausher, 2020), while *CgMYB1* is only associated with the red spot region (Martins et al., 2017). Interestingly, a mutant variant of *CgMYB12*, containing a premature stop codon, produces a truncated protein that lacks the function to initiate pigment synthesis exclusively in the white cup region of the petals (Lin and Rausher, 2020). Mutation of *CgMYB12* indicates that the three R2R3 MYBs are spatially restricted; *CgMYB12* is usually restricted to the basal region of the petal, and its mutation allows for complex bicolour patterns to evolve (Fig. 5B.1; Lin and Rausher, 2020).

In *Mimulus*, two disparate pigment classes are associated with complex colour patterns and their evolution. Zygomorphic *Mimulus*, similar to *A. majus*, has two dorsally positioned petals, two lateral petals and one ventral petal. In *Mimulus lewisii*, petals exhibit a bicolour pattern, with a white corolla tube and pink lobes, except for the single ventral petal, which has two overlaying ridges pigmented with carotenoids and speckled with fine anthocyanic red spots. Production of pink flavonoids in petal lobes is associated with the R2R3 MYB, PETAL LOBE ANTHOCYANIN (MlPELAN), while carotenoid production is regulated by another R2R3 MYB, REDUCED CAROTENOID PIGMENTATION 1 (MlRCP1) (Yuan et al., 2014). Overexpression of *RCP1* was shown to repress MlPELAN and likely restricts it in the ventral petal lobes. Simultaneously, MlRCP1 suppresses *YELLOW UPPER* (*YUP*, a non-coding locus containing siRNAs), leading to carotenoid accumulation (Stanley et al., 2020). *MlYUP* silences *MlRCP2*, which encodes an enzyme that regulates carotenoid production indirectly, altering the shape of chromoplasts to disturb carotenoid accumulation (Stanley et al., 2020). Meanwhile, the finely speckled red spots on the carotenoid background are formed by a reaction-diffusion gradient comprised of activation by an R2R3 MYB, NECTAR GUIDE ANTHOCYANIN (MlNEGAN) and its R3 MYB repressor Red Tongue (RTO), which periodically and through autocatalysis produce spots (Fig. 5B.2; Yuan et al., 2014; Ding et al., 2020; Zheng et al., 2021). Occurrence of *YUP*, *PELAN* and another flavonoid regulating R2R2 MYB (*SOLAR*) at the same superlocus suggests that functional selection of the locus and its subsequent tinkering is associated with phenotypic diversity and pollinator shifts in *Mimulus* (Liang et al., 2023).

## Complex flowers as modular developmental systems

Floral displays are modular reproductive structures and the examples above illustrate how variation within each of the four primary developmental modules can generate phenotypic complexity. In this Review, we define floral displays produced by modifying one of these modules as having ‘intermediate complexity’. However, more elaborate complexity can arise when variation occurs in two or more of these modules. As stated above, we define these cases as examples of ‘high complexity’. For example, flowers of *Nigella* (Ranunculaceae) illustrate complexity in organ identity and number, and in pigmentation of the showy perianth (Zhang et al., 2020; Dönmez et al., 2021). Specifically, a typical *Nigella orientalis* produces either solitary flowers or a simple raceme with a few flowers, each comprised of five sepals, eight petals, 30-35 stamens occurring in eight groups, and 4-7 carpels (Dönmez et al., 2021). These variations in organ number and size have been studied in the closely related *Nigella damascena*, where they have been attributed to the size of the FM (Wang et al., 2015). Furthermore, several gene duplications of ABCE genes have been shown in the species, with a feedback loop between B-class NdAP3 and C-class NdAG1 responsible for boundary establishment between petals and stamens (Wang et al., 2015). The petals of *N. orientalis* also display a novel and complex petal identity composed of a bilabiate petal with a triangular upper lip and rhombic lower lip. At maturity, the petals also display colour patterning composed of red horizontal stripes and splatters on the upper lip, which gradually transition to yellow distal regions (Yuan et al., 2023). These complex colour patterns have been attributed to developmental co-option of flavonoid and carotenoid biosynthetic pathways, combined with sub-functionalisation of two R2R3 MYBs (i.e. NiorMYB113-1 and NiorMYB113-2) (Yuan et al., 2023). Specifically, NiorMYB113-1 promotes red pigmentation for the stripe, while NiorMYB113-2 restricts it in the green regions of the flower, allowing carotenoid pigmentation (Yuan et al., 2023). Together, these complex petals provide an elaborate visual cue with their novel petal morphology and pigment patterning. They also exhibit structural modifications to the epidermis (Yao et al., 2019) and specialised structures such as the nectaries (Liao et al., 2020).

Combinations of complexity in inflorescence architecture and flower symmetry are repeatedly observed amongst diverse lineages of asterids. For example, the reduction of the internode lengths between flowers gave rise to the compressed inflorescence, as in the case of the Asteraceae flower head, with flowers emerging in a spiral pattern and all but simultaneously. Within Asteraceae, three distinct capitula types exist: discoid (only actinomorphic disc florets), ligulate (only zygomorphic ray florets) and radiate (both disc and ray florets). In radiate capitula, flowers may obtain distinct identities, with those furthest from the inflorescence axis largely functioning in pollinator attraction, while those in the centre mainly function for reproduction (Elomaa et al., 2018). Further complexity may be added by intermediate florets forming in a ring between the disc and ray florets, presenting zygomorphy but with reduced petal growth, such as seen in *Dahlia* or *Gerbera*. Colour may also differ between floret types, with differential regulation of pigment in ray and disc florets contributing to the overall impression of a single large flower, such as in common garden daisies (Zhang and Elomaa, 2024). Development of the inflorescence and of floral symmetry in representative Asteraceae species have been discussed above. To add further complexity, eight E-class SEPALLATA-like homologues, called the GERBERA REGULATOR OF CAPITULUM DEVELOPMENT genes (GRCDs), likely regulate inflorescence meristem determinacy as well as organ identity (Elomaa et al., 2018). Unlike their *Arabidopsis* orthologues, which share redundant functions in organ identity, GRCDs in *Gerbera* have sub- and neo-functionalised to determine meristem identity, and organ identity, of petals, stamen and carpels (Kotilainen et al., 2000; Uimari et al., 2004; Zhang et al., 2017a,b; Elomaa et al., 2018). Little is known about how these genes interact with *LFY* and *CYC* homologs to specify the distinct identities and symmetry of flowers on the capitula, although all three groups of genes show overlapping expression patterns in ray florets of *Gerbera* (Juntheikki-Palovaara et al., 2014; Zhao et al., 2016; Elomaa et al., 2018).

One of the most common combinations of modules in generating complex flowers is variation in flower symmetry and colour. Since the first discoveries of symmetry development in *Antirrhinum*, it has been speculated that the regulation of flower colour is downstream of the symmetry GRN (Yang et al., 2023). Changes in both flower symmetry and colour have been shown in some cases to be associated with specialisation in animal pollinator groups (Fenster et al., 2004; Martínez-Gómez et al., 2023). Complex symmetry and pigmentation patterns are currently being explored across diverse lineages such as *Antirrhinum*, *Chirita*, *Chrysanthemum* and *Mimulus.* Together, these studies have shown *CYC*-like genes to directly regulate pigmentation by targeting genes encoding biosynthetic enzymes (Zhang et al., 2022a; Yang et al., 2023), epidermal cell identities (Perez-Rodriguez et al., 2005) and transcriptional regulators of pigmentation (Su et al., 2017). These studies illustrate the lability of development and the evolutionary flexibility in connecting disparate modules to influence final flower form. The limited number of studies exploring the connections between these modules highlight the need and urgency to integrate these pathways towards a more complete understanding of complex flower development.

## Conclusion

In this Review, we have attempted to reduce decades of flower and inflorescence developmental studies into a unifying definition of the complexity of floral display. We defined complexity as arising through a combination of four major phenotypic characters of floral displays: inflorescence architecture, organ identity, flower symmetry and flower colour. Each phenotypic module is controlled by elaborate and complex GRNs that are still being established across diverse taxa. Some common patterns have emerged through inspection of key regulatory genes in each GRN. These include the frequency of gene duplications followed by neo and sub-functionalisation, the integral roles of silencing partners (such as siRNAs, miRNAs and protein partners), and frequent and labile changes in gene expression and protein interactions. Further information is likely to emerge from genome sequencing of non-model species and investigation of trait regulation through unbiased and organism-level approaches (such as mutagenesis, single-cell transcriptomics and proteomics). Future investigation and comparison of entire gene regulatory networks and flowering systems under a unifying definition of complexity may allow us to disentangle further evolutionary mechanisms associated with complexity and novelty. Understanding the developmental and evolutionary origins of this complexity will be important in understanding the developmental robustness of flowers and the evolutionary lability of flowering plant lineages, which are both likely to be important in maintaining plant-pollinator networks in the face of changing climates.

## Supplementary Material

10.1242/develop.203027_sup1 Supplementary information

## References

[DEV203027C1] Abad, U., Sassi, M. and Traas, J. (2017). Flower development: from morphodynamics to morphomechanics. *Philos. Trans. R. Soc. B Biol. Sci.* 372, 20150545. 10.1098/rstb.2015.0545 PMC5379030 28348258

[DEV203027C2] Adami, C., Ofria, C. and Collier, T. C. (2000). Evolution of biological complexity. *Proc. Natl Acad. Sci. USA* 97, 4463-4468. 10.1073/pnas.97.9.4463 10781045 PMC18257

[DEV203027C3] Albert, N. W., Butelli, E., Moss, S. M. A., Piazza, P., Waite, C. N., Schwinn, K. E., Davies, K. M. and Martin, C. (2021). Discrete bHLH transcription factors play functionally overlapping roles in pigmentation patterning in flowers of *Antirrhinum majus*. *New Phytol.* 231, 849-863. 10.1111/nph.17142 33616943 PMC8248400

[DEV203027C4] Almeida, J., Rocheta, M. and Galego, L. (1997). Genetic control of flower shape in *Antirrhinum majus*. *Development* 124, 1387-1392. 10.1242/dev.124.7.1387 9118809

[DEV203027C5] Appleton, A. D. and Kramer, E. M. (2024). Genetic architecture of novel floral organs. *Int. J. Plant Sci.* 185, 211-217. 10.1086/729359

[DEV203027C7] Bačovský, V., Čegan, R., Tihlaříková, E., Neděla, V., Hudzieczek, V., Smrža, L., Beneš, V. and Hobza, R. (2021). Identification of developmentally important genes in *Silene latifolia* through chemical genetics and transcriptome profiling. *bioRxiv*. 10.1101/2021.01.25.428076 35045170

[DEV203027C8] Ballerini, E. S., Min, Y., Edwards, M. B., Kramer, E. M. and Hodges, S. A. (2020). POPOVICH, encoding a C2H2 zinc-finger transcription factor, plays a central role in the development of a key innovation, floral nectar spurs, in Aquilegia. *Proc. Natl Acad. Sci. USA* 117, 22552-22560. 10.1073/pnas.2006912117 32848061 PMC7486772

[DEV203027C9] Bateman, R. M., Hilton, J. and Rudall, P. J. (2006). Morphological and molecular phylogenetic context of the angiosperms: contrasting the ‘top-down‘ and ‘bottom-up‘ approaches used to infer the likely characteristics of the first flowers. *J. Exp. Bot.* 57, 3471-3503. 10.1093/jxb/erl128 17056677

[DEV203027C10] Benlloch, R., Berbel, A., Serrano-Mislata, A. and Madueño, F. (2007). Floral initiation and inflorescence architecture: a comparative view. *Ann. Bot.* 100, 659-676. 10.1093/aob/mcm146 17679690 PMC2533596

[DEV203027C11] Berger, B. A., Thompson, V., Lim, A., Ricigliano, V. and Howarth, D. G. (2016). Elaboration of bilateral symmetry across *Knautia macedonica* capitula related to changes in ventral petal expression of CYCLOIDEA-like genes. *EvoDevo* 7, 1-10. 10.1186/s13227-016-0045-7 27042288 PMC4818532

[DEV203027C12] Bevan, M., Mayer, K., White, O., Eisen, J. A., Preuss, D., Bureau, T., Salzberg, S. L. and Mewes, H.-W. (2001). Sequence and analysis of the *Arabidopsis* genome. *Curr. Opin. Plant Biol.* 4, 105-110. 10.1016/S1369-5266(00)00144-8 11228431

[DEV203027C14] Bohs, L., Weese, T., Myers, N., Lefgren, V., Thomas, N., Van Wagenen, A. and Stern, S. (2007). Zygomorphy and heteranthery in Solanum in a phylogenetic context. *Acta Horticulturae* 745, 201. 10.17660/ActaHortic.2007.745.8

[DEV203027C15] Bowman, J. L. and Floyd, S. K. (2008). Patterning and polarity in seed plant shoots. *Annu. Rev. Plant Biol.* 59, 67-88. 10.1146/annurev.arplant.57.032905.105356 18031217

[DEV203027C16] Bowman, J. L., Smyth, D. R. and Meyerowitz, E. M. (1989). Genes directing flower development in Arabidopsis. *Plant Cell* 1, 37-52. 2535466 10.1105/tpc.1.1.37 PMC159735

[DEV203027C17] Bowman, J. L., Smyth, D. R. and Meyerowitz, E. M. (1991). Genetic interactions among floral homeotic genes of Arabidopsis. *Development* 112, 1-20. 10.1242/dev.112.1.1 1685111

[DEV203027C18] Bradley, D., Carpenter, R., Copsey, L., Vincent, C., Rothstein, S. and Coen, E. (1996). Control of inflorescence architecture in Antirrhinum. *Nature* 379, 791-797. 10.1038/379791a0 8587601

[DEV203027C19] Bradley, D., Ratcliffe, O., Vincent, C., Carpenter, R. and Coen, E. (1997). Inflorescence commitment and architecture in *Arabidopsis*. *Science* 275, 80-83. 10.1126/science.275.5296.80 8974397

[DEV203027C20] Bradley, D., Xu, P., Mohorianu, I.-I., Whibley, A., Field, D., Tavares, H., Couchman, M., Copsey, L., Carpenter, R. and Li, M. (2017). Evolution of flower colour pattern through selection on regulatory small RNAs. *Science* 358, 925-928. 10.1126/science.aao3526 29146812

[DEV203027C21] Broholm, S. K., Tähtiharju, S., Laitinen, R. A. E., Albert, V. A., Teeri, T. H. and Elomaa, P. (2008). A TCP domain transcription factor controls flower type specification along the radial axis of the Gerbera (Asteraceae) inflorescence. *Proc. Natl. Acad. Sci. U.S.A.* 105, 9117-9122. 10.1073/pnas.0801359105 18574149 PMC2449374

[DEV203027C22] Bukhari, G., Zhang, J., Stevens, P. F. and Zhang, W. (2017). Evolution of the process underlying floral zygomorphy development in pentapetalous angiosperms. *Am. J. Bot.* 104, 1846-1856. 10.3732/ajb.1700229 29247025

[DEV203027C23] Burleigh, J. G., Whittall, J. B. and Sanderson, M. J. (2006). The Evolution of Organismal Complexity in Angiosperms as Measured by the Information Content of Taxonomic Descriptions. Workshop Proceedings of the Tenth International Conference on the Simulation and Synthesis of Living Systems, MIT Press, pp. 87-92.

[DEV203027C24] Carlson, S. E., Howarth, D. G. and Donoghue, M. J. (2011). Diversification of CYCLOIDEA-like genes in Dipsacaceae (Dipsacales): implications for the evolution of capitulum inflorescences. *BMC Evol. Biol.* 11, 1-13. 10.1186/1471-2148-11-325 22054400 PMC3224765

[DEV203027C25] Chae, E., Tan, Q. K.-G., Hill, T. A. and Irish, V. F. (2008). An *Arabidopsis* F-box protein acts as a transcriptional co-factor to regulate floral development. *Development* 135, 1235-1245. 10.1242/dev.015842 18287201

[DEV203027C26] Chapman, M. A., Tang, S., Draeger, D., Nambeesan, S., Shaffer, H., Barb, J. G., Knapp, S. J. and Burke, J. M. (2012). Genetic analysis of floral symmetry in Van Gogh‘s sunflowers reveals independent recruitment of CYCLOIDEA genes in the Asteraceae. *PLoS Genet.* 8, e1002628. 10.1371/journal.pgen.1002628 22479210 PMC3315478

[DEV203027C27] Charlesworth, D. (2016). Plant sex chromosomes. *Annu. Rev. Plant Biol.* 67, 397-420. 10.1146/annurev-arplant-043015-111911 26653795

[DEV203027C28] Coen, E. S. and Meyerowitz, E. M. (1991). The war of the whorls: genetic interactions controlling flower development. *Nature* 353, 31-37. 10.1038/353031a0 1715520

[DEV203027C29] Coen, E. S. and Nugent, J. M. (1994). Evolution of flowers and inflorescences. *Development* 1994, 107-116. 10.1242/dev.1994.Supplement.107

[DEV203027C30] Coen, E. and Prusinkiewicz, P. (2024). Developmental timing in plants. *Nat. Commun.* 15, 2674. 10.1038/s41467-024-46941-1 38531864 PMC10965974

[DEV203027C31] Conway, S. J., Walcher-Chevillet, C. L., Salome Barbour, K. and Kramer, E. M. (2021). Brassinosteroids regulate petal spur length in Aquilegia by controlling cell elongation. *Ann. Bot.* 128, 931-942. 10.1093/aob/mcab116 34508638 PMC8577200

[DEV203027C32] Corley, S. B., Carpenter, R., Copsey, L. and Coen, E. (2005). Floral asymmetry involves an interplay between TCP and MYB transcription factors in *Antirrhinum*. *Proc. Natl. Acad. Sci. U.S.A.* 102, 5068-5073. 10.1073/pnas.0501340102 15790677 PMC555980

[DEV203027C33] Costa, M. M. R., Fox, S., Hanna, A. I., Baxter, C. and Coen, E. (2005). Evolution of regulatory interactions controlling floral asymmetry. *Development* 132, 5093-5101. 10.1242/dev.02085 16236768

[DEV203027C34] Davies, B., Cartolano, M. and Schwarz-Sommer, Z. (2006). Flower development: the *Antirrhinum* perspective. *Adv. Bot. Res.* 44, 279-321. 10.1016/S0065-2296(06)44007-6

[DEV203027C35] Ding, B., Patterson, E. L., Holalu, S. V., Li, J., Johnson, G. A., Stanley, L. E., Greenlee, A. B., Peng, F., Bradshaw, H. and Blinov, M. L. (2020). Two MYB proteins in a self-organizing activator-inhibitor system produce spotted pigmentation patterns. *Curr. Biol.* 30, 802-814. 10.1016/j.cub.2019.12.067 32155414 PMC7156294

[DEV203027C36] Dönmez, A. A., Aydin, Z. U. and Dönmez, E. O. (2021). Taxonomic monograph of the tribe Nigelleae (Ranunculaceae): a group including ancient medicinal plants. *Turk. J. Bot.* 45, 468-502. 10.3906/bot-2105-39

[DEV203027C37] Donoghue, M. J., Ree, R. H. and Baum, D. A. (1998). Phylogeny and the evolution of flower symmetry in the Asteridae. *Trends Plant Sci.* 3, 311-317. 10.1016/S1360-1385(98)01278-3

[DEV203027C38] Dreni, L. and Kater, M. M. (2014). MADS reloaded: evolution of the AGAMOUS subfamily genes. *New Phytol.* 201, 717-732. 10.1111/nph.12555 24164649

[DEV203027C39] Edwards, M. B., Ballerini, E. S. and Kramer, E. M. (2022). Complex developmental and transcriptional dynamics underlie pollinator-driven evolutionary transitions in nectar spur morphology in Aquilegia (columbine). *Am. J. Bot.* 109, 1360-1381. 10.1002/ajb2.16046 35971626

[DEV203027C40] Elomaa, P., Zhao, Y. and Zhang, T. (2018). Flower heads in Asteraceae—recruitment of conserved developmental regulators to control the flower-like inflorescence architecture. *Hortic. Res.* 5, 36. 10.1038/s41438-018-0056-8 29977572 PMC6026493

[DEV203027C41] Endress, P. K. (2010). Flower structure and trends of evolution in eudicots and their major subclades. *Ann. Mo. Bot. Gard.* 97, 541-583. 10.3417/2009139

[DEV203027C179] Evert, R. F. and Eichorn, S. E. (2012). *Raven Biology of Plants*, 8th edition. W.H. Freeman.

[DEV203027C43] Fattorini, R., Khojayori, F. N., Mellers, G., Moyroud, E., Herrero, E., Kellenberger, R. T., Walker, R., Wang, Q., Hill, L. and Glover, B. J. (2024). Complex petal spot formation in the Beetle Daisy (*Gorteria diffusa*) relies on spot-specific accumulation of malonylated anthocyanin regulated by paralogous GdMYBSG 6 transcription factors. *New Phytol.* 243, 240-257. 10.1111/nph.19804 38725421

[DEV203027C44] Feng, C. M., Liu, X., Yu, Y., Xie, D., Franks, R. G. and Xiang, Q. Y. (2012). Evolution of bract development and B-class MADS box gene expression in petaloid bracts of Cornus s. l.(Cornaceae). *New Phytol.* 196, 631-643. 10.1111/j.1469-8137.2012.04255.x 22897242

[DEV203027C45] Fenster, C. B., Armbruster, W. S., Wilson, P., Dudash, M. R. and Thomson, J. D. (2004). Pollination syndromes and floral specialization. *Ann. Rev. Ecol. Evol. Syst.* 35, 375-403. 10.1146/annurev.ecolsys.34.011802.132347

[DEV203027C46] Freiberg, M., Winter, M., Gentile, A., Zizka, A., Muellner-Riehl, A. N., Weigelt, A. and Wirth, C. (2020). LCVP, The Leipzig catalogue of vascular plants, a new taxonomic reference list for all known vascular plants. *Sci. Data* 7, 416. 10.1038/s41597-020-00702-z 33243996 PMC7693275

[DEV203027C47] Geuten, K., Becker, A., Kaufmann, K., Caris, P., Janssens, S., Viaene, T., Theißen, G. and Smets, E. (2006). Petaloidy and petal identity MADS-box genes in the balsaminoid genera Impatiens and Marcgravia. *Plant J.* 47, 501-518. 10.1111/j.1365-313X.2006.02800.x 16856983

[DEV203027C48] Gilbert, R. (1971). An unusual anthocyanin in *Antirrhinum majus*. *Phytochemistry* 10, 2848-2849. 10.1016/S0031-9422(00)97309-6

[DEV203027C49] Gompel, N. and Prud‘homme, B. (2009). The causes of repeated genetic evolution. *Dev. Biol.* 332, 36-47. 10.1016/j.ydbio.2009.04.040 19433086

[DEV203027C50] Gorton, H. L. and Vogelmann, T. C. (1996). Effects of epidermal cell shape and pigmentation on optical properties of *Antirrhinum* petals at visible and ultraviolet wavelengths. *Plant Physiol.* 112, 879-888. 10.1104/pp.112.3.879 12226425 PMC158014

[DEV203027C51] Green, A. A., Kennaway, J. R., Hanna, A. I., Bangham, J. A. and Coen, E. (2010). Genetic control of organ shape and tissue polarity. *PLoS Biol.* 8, e1000537. 10.1371/journal.pbio.1000537 21085690 PMC2976718

[DEV203027C52] Grotewold, E. (2006). *The Science of Flavonoids*: Springer.

[DEV203027C53] Hardenack, S., Ye, D., Saedler, H. and Grant, S. (1994). Comparison of MADS box gene expression in developing male and female flowers of the dioecious plant white campion. *Plant Cell* 6, 1775-1787. 7866023 10.1105/tpc.6.12.1775 PMC160561

[DEV203027C55] Honma, T. and Goto, K. (2001). Complexes of MADS-box proteins are sufficient to convert leaves into floral organs. *Nature* 409, 525-529. 10.1038/35054083 11206550

[DEV203027C56] Howarth, D. G. and Donoghue, M. J. (2006). Phylogenetic analysis of the “ECE” (CYC/TB1) clade reveals duplications predating the core eudicots. *Proc. Natl. Acad. Sci. U.S.A.* 103, 9101-9106. 10.1073/pnas.0602827103 16754863 PMC1482573

[DEV203027C57] Hsu, H.-F., Hsu, W.-H., Lee, Y.-I., Mao, W.-T., Yang, J.-Y., Li, J.-Y. and Yang, C.-H. (2015). Model for perianth formation in orchids. *Nat. Plants* 1, 1-8.

[DEV203027C58] Ikeda, K., Ito, M., Nagasawa, N., Kyozuka, J. and Nagato, Y. (2007). Rice ABERRANT PANICLE ORGANIZATION 1, encoding an F-box protein, regulates meristem fate. *Plant J.* 51, 1030-1040. 10.1111/j.1365-313X.2007.03200.x 17666027

[DEV203027C59] Ikeda, T., Tanaka, W., Toriba, T., Suzuki, C., Maeno, A., Tsuda, K., Shiroishi, T., Kurata, T., Sakamoto, T. and Murai, M. (2019). BELL 1-like homeobox genes regulate inflorescence architecture and meristem maintenance in rice. *Plant J.* 98, 465-478. 10.1111/tpj.14230 30657229

[DEV203027C60] Ikeda-Kawakatsu, K., Yasuno, N., Oikawa, T., Iida, S., Nagato, Y., Maekawa, M. and Kyozuka, J. (2009). Expression level of ABERRANT PANICLE ORGANISATION 1 determines rice inflorescence form through control of cell proliferation in the meristem. *Plant Physiol.* 150, 736-747. 10.1104/pp.109.136739 19386809 PMC2689948

[DEV203027C61] Irish, V. F. (2010). The flowering of *Arabidopsis* flower development. *Plant J.* 61, 1014-1028. 10.1111/j.1365-313X.2009.04065.x 20409275

[DEV203027C62] Jain, B. and Pandey, S. (2018). WD40 repeat proteins: signalling scaffold with diverse functions. *Protein J.* 37, 391-406. 10.1007/s10930-018-9785-7 30069656

[DEV203027C63] Juntheikki-Palovaara, I., Tahtiharju, S., Lan, T., Broholm, S., Rijpkema, A., Ruonola, R., Kale, L., Albert, V., Teeri, T. and Elomaa, P. (2014). Functional diversification of duplicated CYC2 clade genes in regulation of inflorescence development in *Gerbera hybrida* (Asteraceae). *Plant J.* 79, 783-796. 10.1111/tpj.12583 24923429

[DEV203027C64] Käfer, J., Marais, G. A. and Pannell, J. R. (2017). On the rarity of dioecy in flowering plants. *Mol. Ecol.* 26, 1225-1241. 10.1111/mec.14020 28101895

[DEV203027C65] Kater, M. M., Dreni, L. and Colombo, L. (2006). Functional conservation of MADS-box factors controlling floral organ identity in rice and *Arabidopsis*. *J. Exp. Bot.* 57, 3433-3444. 10.1093/jxb/erl097 16968881

[DEV203027C66] Kellenberger, R. T. and Glover, B. J. (2023). The evolution of flower colour. *Curr. Biol.* 33, R484-R488. 10.1016/j.cub.2023.01.055 37279680

[DEV203027C67] Kellenberger, R. T., Ponraj, U., Delahaie, B., Fattorini, R., Balk, J., Lopez-Gomollon, S., Müller, K. H., Ellis, A. G. and Glover, B. J. (2023). Multiple gene co-options underlie the rapid evolution of sexually deceptive flowers in Gorteria diffusa. *Curr. Biol.* 33, 1502-1512. 10.1016/j.cub.2023.03.003 36963385

[DEV203027C68] Kim, M., Cui, M.-L., Cubas, P., Gillies, A., Lee, K., Chapman, M. A., Abbott, R. J. and Coen, E. (2008). Regulatory genes control a key morphological and ecological trait transferred between species. *Science* 322, 1116-1119. 10.1126/science.1164371 19008450

[DEV203027C69] Knapp, S. (2002). Floral diversity and evolution in the Solanaceae. In *Developmental Genetics and Plant Evolution*, pp. 267-297: Taylor and Francis.

[DEV203027C70] Knapp, S. (2010). On ‘various contrivances’: pollination, phylogeny and flower form in the Solanaceae. *Philos. Trans. R. Soc. B Biol. Sci.* 365, 449-460. 10.1098/rstb.2009.0236 PMC2838263 20047871

[DEV203027C71] Koes, R., Verweij, W. and Quattrocchio, F. (2005). Flavonoids: a colourful model for the regulation and evolution of biochemical pathways. *Trends Plant Sci.* 10, 236-242. 10.1016/j.tplants.2005.03.002 15882656

[DEV203027C72] Kotilainen, M., Elomaa, P., Uimari, A., Albert, V. A., Yu, D. and Teeri, T. H. (2000). GRCD1, an AGL2-like MADS box gene, participates in the C function during stamen development in *Gerbera hybrida*. *Plant Cell* 12, 1893-1902. 10.1105/tpc.12.10.1893 11041884 PMC149127

[DEV203027C73] Kramer, E. M. (2009). Aquilegia: a new model for plant development, ecology, and evolution. *Annu. Rev. Plant Biol.* 60, 261-277. 10.1146/annurev.arplant.043008.092051 19575583

[DEV203027C74] Kramer, E. M. and Jaramillo, M. A. (2005). Genetic basis for innovations in floral organ identity. *J. Exp. Zool. B Mol. Dev. Evol.* 304, 526-535. 10.1002/jez.b.21046 15880769

[DEV203027C75] Krishna, S. and Keasar, T. (2018). Morphological complexity as a floral signal: from perception by insect pollinators to co-evolutionary implications. *Int. J. Mol. Sci.* 19, 1681. 10.3390/ijms19061681 29882762 PMC6032408

[DEV203027C76] Kyozuka, J., Konishi, S., Nemoto, K., Izawa, T. and Shimamoto, K. (1998). Down-regulation of RFL, the FLO/LFY homolog of rice, accompanied with panicle branch initiation. *Proc. Natl Acad. Sci. USA* 95, 1979-1982. 10.1073/pnas.95.5.1979 9482818 PMC33826

[DEV203027C77] Lee, I., Wolfe, D. S., Nilsson, O. and Weigel, D. (1997). A leafy co-regulator encoded by unusual floral organs. *Curr. Biol.* 7, 95-104. 10.1016/S0960-9822(06)00053-4 9016705

[DEV203027C78] Leslie, A. B. and Mander, L. (2023). Quantifying the complexity of plant reproductive structures reveals a history of morphological and functional integration. *Proc. R. Soc. B* 290, 20231810. 10.1098/rspb.2023.1810 PMC10618862 37909082

[DEV203027C79] Liang, M., Chen, W., LaFountain, A. M., Liu, Y., Peng, F., Xia, R., Bradshaw, H. and Yuan, Y.-W. (2023). Taxon-specific, phased siRNAs underlie a speciation locus in monkeyflowers. *Science* 379, 576-582. 10.1126/science.adf1323 36758083 PMC10601778

[DEV203027C80] Liao, H., Fu, X., Zhao, H., Cheng, J., Zhang, R., Yao, X., Duan, X., Shan, H. and Kong, H. (2020). The morphology, molecular development and ecological function of pseudonectaries on *Nigella damascena* (Ranunculaceae) petals. *Nat. Commun.* 11, 1777. 10.1038/s41467-020-15658-2 32286317 PMC7156421

[DEV203027C81] Lin, R.-C. and Rausher, M. D. (2020). R2R3-MYB genes control petal pigmentation patterning in *Clarkia gracilis ssp. sonomensis* (Onagraceae). *New Phytol.* 229, 1147-1162. 10.1111/nph.16908 32880946

[DEV203027C82] Lin, R.-C. and Rausher, M. D. (2021). Ancient gene duplications, rather than polyploidization, facilitate diversification of petal pigmentation patterns in *Clarkia gracilis* (Onagraceae). *Mol. Biol. Evol.* 38, 5528-5538. 10.1093/molbev/msab242 34398232 PMC8662608

[DEV203027C83] Litt, A. and Kramer, E. (2010). The ABC model and the diversification of floral organ identity. *Semin. Cell Dev. Biol.* 21, 129-137. 10.1016/j.semcdb.2009.11.019 19948236

[DEV203027C84] Lopez-Garcia, M., Masters, N., O‘Brien, H. E., Lennon, J., Atkinson, G., Cryan, M. J., Oulton, R. and Whitney, H. M. (2018). Light-induced dynamic structural colour by intracellular 3D photonic crystals in brown algae. *Sci. Adv.* 4, eaan8917. 10.1126/sciadv.aan8917 29651457 PMC5895443

[DEV203027C85] López-Martínez, A. M., Magallón, S., von Balthazar, M., Schönenberger, J., Sauquet, H. and Chartier, M. (2024). Angiosperm flowers reached their highest morphological diversity early in their evolutionary history. *New Phytol.* 241, 1348-1360. 10.1111/nph.19389 38029781 PMC10952840

[DEV203027C86] Luo, D., Carpenter, R., Vincent, C., Copsey, L. and Coen, E. (1996). Origin of floral asymmetry in *Antirrhinum*. *Nature* 383, 794-799. 10.1038/383794a0 8893002

[DEV203027C87] Martin, A. and Orgogozo, V. (2013). The loci of repeated evolution: a catalog of genetic hotspots of phenotypic variation. *Evolution* 67, 1235-1250. 23617905 10.1111/evo.12081

[DEV203027C88] Martin, C., Prescott, A., Mackay, S., Bartlett, J. and Vrijlandt, E. (1991). Control of anthocyanin biosynthesis in flowers of *Antirrhinum majus*. *Plant J.* 1, 37-49. 10.1111/j.1365-313X.1991.00037.x 1844879

[DEV203027C89] Martins, T., Jiang, P. and Rausher, M. (2017). How petals change their spots: cis-regulatory rewiring in *Clarkia* (Onagraceae). *New Phytol.* 216, 510-518. 10.1111/nph.14163 27597114

[DEV203027C90] Martínez-Gómez, J., Park, S., Hartogs, S. R., Soza, V. L., Park, S. J. and Di Stilio, V. S. (2023). Flower morphology as a predictor of pollination mode in a biotic to abiotic pollination continuum. *Ann. Bot.* 132, 61-76. 10.1093/aob/mcad069 37235981 PMC10550269

[DEV203027C91] Meyerowitz, E. M. (2001). Prehistory and history of *Arabidopsis* research. *Plant Physiol.* 125, 15-19. 10.1104/pp.125.1.15 11154286 PMC1539315

[DEV203027C92] Moss, S. M. A., Zhou, Y., Butelli, E., Waite, C. N., Yeh, S.-M., Cordiner, S. B., Harris, N. N., Copsey, L., Schwinn, K. E., Davies, K. M. et al. (2024). Painted flowers: *Eluta* generates pigment patterning in *Antirrhinum*. *New Phytol.* 243, 738-752. 10.1111/nph.19866 38822654

[DEV203027C93] Moyroud, E., Kusters, E., Monniaux, M., Koes, R. and Parcy, F. (2010). LEAFY blossoms. *Trends Plant Sci.* 15, 346-352. 10.1016/j.tplants.2010.03.007 20413341

[DEV203027C94] Moyroud, E., Wenzel, T., Middleton, R., Rudall, P. J., Banks, H., Reed, A., Mellers, G., Killoran, P., Westwood, M. M., Steiner, U. et al. (2017). Disorder in convergent floral nanostructures enhances signalling to bees. *Nature* 550, 469-474. 10.1038/nature24285 29045384

[DEV203027C95] Nakayama, T. (2002). Enzymology of aurone biosynthesis. *J. Biosci. Bioeng.* 94, 487-491. 10.1016/S1389-1723(02)80184-0 16233339

[DEV203027C96] Narbona, E., del Valle, J. C., Arista, M., Buide, M. L. and Ortiz, P. L. (2021). Major flower pigments originate different colour signals to pollinators. *Front. Ecol. Evol.* 9, 743850. 10.3389/fevo.2021.743850

[DEV203027C97] Noda, K.-I., Glover, B. J., Linstead, P. and Martin, C. (1994). Flower colour intensity depends on specialized cell shape controlled by a Myb-related transcription factor. *Nature* 369, 661-664. 10.1038/369661a0 8208293

[DEV203027C98] Parcy, F. (2005). Flowering: a time for integration. *Int. J. Dev. Biol.* 49, 585. 10.1387/ijdb.041930fp 16096967

[DEV203027C99] Pavličev, M. and Cheverud, J. M. (2015). Constraints evolve: context dependency of gene effects allows evolution of pleiotropy. *Ann. Rev. Ecol. Evol. Syst.* 46, 413-434. 10.1146/annurev-ecolsys-120213-091721

[DEV203027C100] Paz-Ares, J., Ghosal, D., Wienand, U., Peterson, P. and Saedler, H. (1987). The regulatory c1 locus of *Zea mays* encodes a protein with homology to myb proto-oncogene products and with structural similarities to transcriptional activators. *EMBO J.* 6, 3553-3558. 10.1002/j.1460-2075.1987.tb02684.x 3428265 PMC553820

[DEV203027C101] Pelaz, S., Ditta, G. S., Baumann, E., Wisman, E. and Yanofsky, M. F. (2000). B and C floral organ identity functions require SEPALLATA MADS-box genes. *Nature* 405, 200-203. 10.1038/35012103 10821278

[DEV203027C102] Perez-Rodriguez, M., Jaffe, F. W., Butelli, E., Glover, B. J. and Martin, C. (2005). Development of three different cell types is associated with the activity of a specific MYB transcription factor in the ventral petal of *Antirrhinum majus* flowers. *Development* 132, 359-370. 10.1242/dev.01584 15604096

[DEV203027C104] Puzey, J. R., Gerbode, S. J., Hodges, S. A., Kramer, E. M. and Mahadevan, L. (2012). Evolution of spur-length diversity in Aquilegia petals is achieved solely through cell-shape anisotropy. *Proc. R. Soc. B* 279, 1640-1645. 10.1098/rspb.2011.1873 PMC3282339 22090381

[DEV203027C105] Qian, H., Zhang, J. and Zhao, J. (2022). How many known vascular plant species are there in the world? An integration of multiple global plant databases. *Biodivers. Sci.* 30, 22254. 10.17520/biods.2022254

[DEV203027C106] Quattrocchio, F., Wing, J., van der Woude, K., Souer, E., de Vetten, N., Mol, J. and Koes, R. (1999). Molecular analysis of the anthocyanin2 gene of petunia and its role in the evolution of flower color. *Plant Cell* 11, 1433-1444. 10.1105/tpc.11.8.1433 10449578 PMC144295

[DEV203027C107] Raimundo, J., Sobral, R., Bailey, P., Azevedo, H., Galego, L., Almeida, J., Coen, E. and Costa, M. (2013). A subcellular tug of war involving three MYB-like proteins underlies a molecular antagonism in Antirrhinum floral asymmetry. *Plant J.* 75, 527-538. 10.1111/tpj.12225 23638688

[DEV203027C108] Ramsay, N. A. and Glover, B. J. (2005). MYB–bHLH–WD40 protein complex and the evolution of cellular diversity. *Trends Plant Sci.* 10, 63-70. 10.1016/j.tplants.2004.12.011 15708343

[DEV203027C109] Rao, N. N., Prasad, K., Kumar, P. R. and Vijayraghavan, U. (2008). Distinct regulatory role for RFL, the rice LFY homolog, in determining flowering time and plant architecture. *Proc. Natl Acad. Sci. USA* 105, 3646-3651. 10.1073/pnas.0709059105 18305171 PMC2265177

[DEV203027C111] Rausher, M. D. (2008). Evolutionary transitions in floral colour. *Int. J. Plant Sci.* 169, 7-21. 10.1086/523358

[DEV203027C112] Rebocho, A. B., Kennaway, J. R., Bangham, J. A. and Coen, E. (2017). Formation and shaping of the Antirrhinum flower through modulation of the CUP boundary gene. *Curr. Biol.* 27, 2610-2622. 10.1016/j.cub.2017.07.064 28867204

[DEV203027C178] Remizowa, M. V., Sokoloff, D. D. and Rudall, P. J. (2010). Evolutionary history of the monocot flower. *Ann. Missouri Bot. Gard.* 97, 617-645. 10.3417/2009142

[DEV203027C113] Ren, R., Wang, H., Guo, C., Zhang, N., Zeng, L., Chen, Y., Ma, H. and Qi, J. (2018). Widespread whole genome duplications contribute to genome complexity and species diversity in angiosperms. *Mol. Plant* 11, 414-428. 10.1016/j.molp.2018.01.002 29317285

[DEV203027C114] Renner, S. S. (2014). The relative and absolute frequencies of angiosperm sexual systems: dioecy, monoecy, gynodioecy, and an updated online database. *Am. J. Bot.* 101, 1588-1596. 10.3732/ajb.1400196 25326608

[DEV203027C115] Reyes, E., Sauquet, H. and Nadot, S. (2016). Perianth symmetry changed at least 199 times in angiosperm evolution. *Taxon* 65, 945-964. 10.12705/655.1

[DEV203027C116] Rieu, P., Turchi, L., Thevenon, E., Zarkadas, E., Nanao, M., Chahtane, H., Tichtinsky, G., Lucas, J., Blanc-Mathieu, R., Zubieta, C. et al. (2023). The F-box protein UFO controls flower development by redirecting the master transcription factor LEAFY to new cis-elements. *Nat. Plants* 9, 315-329. 10.1038/s41477-022-01336-2 36732360

[DEV203027C117] Robyns, W. (1931). *L‘Organisation florale des solanacées zygomorphes.* M. Lamertin.

[DEV203027C118] Ronse de Craene, L. R. and Smets, E. (2001). Staminodes: their morphological and evolutionary significance. *Bot. Rev.* 67, 351-402. 10.1007/BF02858099

[DEV203027C119] Sauquet, H., Von Balthazar, M., Magallón, S., Doyle, J. A., Endress, P. K., Bailes, E. J., Barroso De Morais, E., Bull-Hereñu, K., Carrive, L., Chartier, M. et al. (2017). The ancestral flower of angiosperms and its early diversification. *Nat. Commun.* 8 16047. 10.1038/ncomms16047 28763051 PMC5543309

[DEV203027C120] Schwinn, K., Venail, J., Shang, Y., Mackay, S., Alm, V., Butelli, E., Oyama, R., Bailey, P., Davies, K. and Martin, C. (2006). A small family of MYB-regulatory genes controls floral pigmentation intensity and patterning in the genus *Antirrhinum*. *Plant Cell* 18, 831-851. 10.1105/tpc.105.039255 16531495 PMC1425845

[DEV203027C121] Shang, Y., Venail, J., Mackay, S., Bailey, P. C., Schwinn, K. E., Jameson, P. E., Martin, C. R. and Davies, K. M. (2011). The molecular basis for venation patterning of pigmentation and its effect on pollinator attraction in flowers of *Antirrhinum*. *New Phytol.* 189, 602-615. 10.1111/j.1469-8137.2010.03498.x 21039563

[DEV203027C122] Sharma, B. and Kramer, E. (2013). Sub-and neo-functionalization of APETALA 3 paralogues have contributed to the evolution of novel floral organ identity in Aquilegia (columbine, Ranunculaceae). *New Phytol.* 197, 949-957. 10.1111/nph.12078 23278258

[DEV203027C123] Smyth, D. R., Bowman, J. L. and Meyerowitz, E. M. (1990). Early flower development in *Arabidopsis*. *Plant Cell* 2, 755-767. 2152125 10.1105/tpc.2.8.755 PMC159928

[DEV203027C124] Souer, E., Krol, A. v. d., Kloos, D., Spelt, C., Bliek, M., Mol, J. and Koes, R. (1998). Genetic control of branching pattern and floral identity during Petunia inflorescence development. *Development* 125, 733-742. 10.1242/dev.125.4.733 9435293

[DEV203027C125] Souer, E., Rebocho, A. B., Bliek, M., Kusters, E., de Bruin, R. A. and Koes, R. (2008). Patterning of inflorescences and flowers by the F-Box protein DOUBLE TOP and the LEAFY homolog ABERRANT LEAF AND FLOWER of petunia. *Plant Cell* 20, 2033-2048. 10.1105/tpc.108.060871 18713949 PMC2553618

[DEV203027C126] Stanley, L. E., Ding, B., Sun, W., Mou, F., Hill, C., Chen, S. and Yuan, Y.-W. (2020). A tetratricopeptide repeat protein regulates carotenoid biosynthesis and chromoplast development in monkeyflowers (*Mimulus*). *Plant Cell* 32, 1536-1555. 10.1105/tpc.19.00755 32132132 PMC7203930

[DEV203027C127] Streisfeld, M. A. and Rausher, M. D. (2011). Population genetics, pleiotropy, and the preferential fixation of mutations during adaptive evolution. *Evolution* 65, 629-642. 10.1111/j.1558-5646.2010.01165.x 21054357

[DEV203027C128] Su, C.-L., Chen, W.-C., Lee, A.-Y., Chen, C.-Y., Chang, Y.-C. A., Chao, Y.-T. and Shih, M.-C. (2013). A modified ABCDE model of flowering in orchids based on gene expression profiling studies of the moth orchid *Phalaenopsis aphrodite*. *PLoS ONE* 8, e80462. 10.1371/journal.pone.0080462 24265826 PMC3827201

[DEV203027C129] Su, S., Xiao, W., Guo, W., Yao, X., Xiao, J., Ye, Z., Wang, N., Jiao, K., Lei, M. and Peng, Q. (2017). The CYCLOIDEA–RADIALIS module regulates petal shape and pigmentation, leading to bilateral corolla symmetry in *Torenia fournieri* (Linderniaceae). *New Phytol.* 215, 1582-1593. 10.1111/nph.14673 28691160

[DEV203027C130] Sun, J., Bhushan, B. and Tong, J. (2013). Structural colouration in nature. *Rsc Advances* 3, 14862-14889. 10.1039/c3ra41096j

[DEV203027C131] Takhtajan, A. L. (1991). *Evolutionary Trends in Flowering Plants*: Columbia University Press.

[DEV203027C132] Tanaka, Y., Sasaki, N. and Ohmiya, A. (2008). Biosynthesis of plant pigments: anthocyanins, betalains and carotenoids. *Plant J.* 54, 733-749. 10.1111/j.1365-313X.2008.03447.x 18476875

[DEV203027C133] Teo, Z. W. N., Zhou, W. and Shen, L. (2019). Dissecting the function of MADS-box transcription factors in orchid reproductive development. *Front. Plant Sci.* 10, 489272. 10.3389/fpls.2019.01474 PMC6872546 31803211

[DEV203027C134] The Arabidopsis Genome Initiative (2000). Analysis of the genome sequence of the flowering plant *Arabidopsis thaliana*. *Nature* 408, 796-815. 10.1038/35048692 11130711

[DEV203027C135] Theissen, G. and Gramzow, L. (2016). Structure and evolution of plant MADS domain transcription factors. In *Plant Transcription Factors*, pp. 127-138. Elsevier.

[DEV203027C136] Theissen, G. and Melzer, R. (2007). Molecular mechanisms underlying origin and diversification of the angiosperm flower. *Ann. Bot.* 100, 603-619. 10.1093/aob/mcm143 17670752 PMC2533597

[DEV203027C137] Thirumalmurugan, V., Manivannan, K. and Nanthakumar, S. (2020). Genetic diversity in African marigold (*Tagetes erecta* L.) under Vellore conditions. *Plant Arch.* 20, 3896-3899.

[DEV203027C138] Thomas, M. M., Rudall, P. J., Ellis, A. G., Savolainen, V. and Glover, B. J. (2009). Development of a complex floral trait: the pollinator-attracting petal spots of the beetle daisy, *Gorteria diffusa* (Asteraceae). *Am. J. Bot.* 96, 2184-2196. 10.3732/ajb.0900079 21622334

[DEV203027C139] Timoneda, A., Feng, T., Sheehan, H., Walker-Hale, N., Pucker, B., Lopez-Nieves, S., Guo, R. and Brockington, S. (2019). The evolution of betalain biosynthesis in Caryophyllales. *New Phytol.* 224, 71-85. 10.1111/nph.15980 31172524

[DEV203027C140] Uimari, A., Kotilainen, M., Elomaa, P., Yu, D., Albert, V. A. and Teeri, T. H. (2004). Integration of reproductive meristem fates by a SEPALLATA-like MADS-box gene. *Proc. Natl Acad. Sci. USA* 101, 15817-15822. 10.1073/pnas.0406844101 15505223 PMC524820

[DEV203027C142] Vekemans, D., Viaene, T., Caris, P. and Geuten, K. (2012). Transference of function shapes organ identity in the dove tree inflorescence. *New Phytol.* 193, 216-228. 10.1111/j.1469-8137.2011.03915.x 21992614

[DEV203027C143] Vignolini, S., Moyroud, E., Glover, B. J. and Steiner, U. (2013). Analysing photonic structures in plants. *J. R. Soc. Interface* 10, 20130394. 10.1098/rsif.2013.0394 23883949 PMC3758000

[DEV203027C144] Vignolini, S., Moyroud, E., Hingant, T., Banks, H., Rudall, P. J., Steiner, U. and Glover, B. J. (2014). The flower of *Hibiscus trionum* is both visibly and measurably iridescent. *New Phytol.* 205, 97-101. 10.1111/nph.12958 25040014

[DEV203027C145] Wang, P., Liao, H., Zhang, W., Yu, X., Zhang, R., Shan, H., Duan, X., Yao, X. and Kong, H. (2015). Flexibility in the structure of spiral flowers and its underlying mechanisms. *Nat. Plants* 2, 1-10. 10.1038/nplants.2015.188 27250746

[DEV203027C146] Weberling, F. (1989). Structure and evolutionary tendencies of inflorescences in the Leguminosae. *Monogr. Syst. Bot. Missouri Bot. Garden* 29, 35-58.

[DEV203027C148] Weigel, D. and Nilsson, O. (1995). A developmental switch sufficient for flower initiation in diverse plants. *Nature* 377, 495-500. 10.1038/377495a0 7566146

[DEV203027C149] Weigel, D., Alvarez, J., Smyth, D. R., Yanofsky, M. F. and Meyerowitz, E. M. (1992). LEAFY controls floral meristem identity in *Arabidopsis*. *Cell* 69, 843-859. 10.1016/0092-8674(92)90295-N 1350515

[DEV203027C150] Whibley, A. C., Langlade, N. B., Andalo, C., Hanna, A. I., Bangham, A., Thébaud, C. and Coen, E. (2006). Evolutionary paths underlying flower colour variation in *Antirrhinum*. *Science* 313, 963-966. 10.1126/science.1129161 16917061

[DEV203027C151] Whitney, H. M., Kolle, M., Andrew, P., Chittka, L., Steiner, U. and Glover, B. J. (2009). Floral Iridescence, produced by diffractive optics, acts as a cue for animal pollinators. *Science* 323, 130-133. 10.1126/science.1166256 19119235

[DEV203027C152] Wilts, B. D., Rudall, P. J., Moyroud, E., Gregory, T., Ogawa, Y., Vignolini, S., Steiner, U. and Glover, B. J. (2018). Ultrastructure and optics of the prism-like petal epidermal cells of *Eschscholzia californica* (California poppy). *New Phytol.* 219, 1124-1133. 10.1111/nph.15229 29856474 PMC6055853

[DEV203027C153] Wolf, Y. I., Katsnelson, M. I. and Koonin, E. V. (2018). Physical foundations of biological complexity. *Proc. Natl Acad. Sci. USA* 115, E8678-E8687. 30150417 10.1073/pnas.1807890115 PMC6140470

[DEV203027C154] Wolkenhauer, O. and Muir, A. (2011). The complexity of cell-biological systems. *Philos. Complex Syst.* 10, 355-385. 10.1016/B978-0-444-52076-0.50013-4

[DEV203027C155] Woodward, A. W. and Bartel, B. (2018). Biology in bloom: a primer on the *Arabidopsis thaliana* model system. *Genetics* 208, 1337-1349. 10.1534/genetics.118.300755 29618591 PMC5887134

[DEV203027C156] Yang, X., Wang, Y., Liu, T.-X., Liu, Q., Liu, J., Lü, T.-F., Yang, R.-X., Guo, F.-X. and Wang, Y.-Z. (2023). CYCLOIDEA-like genes control floral symmetry, floral orientation, and nectar guide patterning. *Plant Cell* 35, 2799-2820. 10.1093/plcell/koad115 37132634 PMC10396386

[DEV203027C157] Yant, L., Collani, S., Puzey, J., Levy, C. and Kramer, E. M. (2015). Molecular basis for three-dimensional elaboration of the Aquilegia petal spur. *Proc. R. Soc. B* 282, 20142778. 10.1098/rspb.2014.2778 PMC4345449 25673682

[DEV203027C158] Yao, X., Zhang, W., Duan, X., Yuan, Y., Zhang, R., Shan, H. and Kong, H. (2019). The making of elaborate petals in Nigella through developmental repatterning. *New Phytol.* 223, 385-396. 10.1111/nph.15799 30889278

[DEV203027C159] Yoshida, H. and Nagato, Y. (2011). Flower development in rice. *J. Exp. Bot.* 62, 4719-4730. 10.1093/jxb/err272 21914655

[DEV203027C160] Yoshida, K., Kitahara, S., Ito, D. and Kondo, T. (2006). Ferric ions involved in the flower colour development of the Himalayan blue poppy, *Meconopsis grandis*. *Phytochemistry* 67, 992-998. 10.1016/j.phytochem.2006.03.013 16678868

[DEV203027C161] Yuan, Y. W., Sagawa, J. M., Frost, L., Vela, J. P. and Bradshaw, H. D., Jr. (2014). Transcriptional control of floral anthocyanin pigmentation in monkeyflowers (Mimulus). *New Phytol.* 204, 1013-1027. 10.1111/nph.12968 25103615 PMC4221532

[DEV203027C162] Yuan, Y. W., Rebocho, A., Sagawa, J., Stanley, L. and Bradshaw, H. (2016). Competition between anthocyanin and favonol biosynthesis produces spatial pattern variation of floral pigments between Mimulus species. *Proc. Natl. Acad. Sci. USA* 107, 6388-6393. 10.1073/pnas.1515294113 PMC4780602 26884205

[DEV203027C163] Yuan, Y., Li, X., Yao, X., Fu, X., Cheng, J., Shan, H., Yin, X. and Kong, H. (2023). Mechanisms underlying the formation of complex colour patterns on *Nigella orientalis* (Ranunculaceae) petals. *New Phytol.* 237, 2450-2466. 10.1111/nph.18681 36527229

[DEV203027C164] Zhang, T. and Elomaa, P. (2024). Development and evolution of the Asteraceae capitulum. *New Phytol.* 242, 33-48. 10.1111/nph.19590 38361269

[DEV203027C165] Zhang, W., Kramer, E. M. and Davis, C. C. (2010). Floral symmetry genes and the origin and maintenance of zygomorphy in a plant-pollinator mutualism. *Proc. Natl. Acad. Sci. U.S.A.* 107, 6388-6393. 10.1073/pnas.0910155107 20363959 PMC2851953

[DEV203027C166] Zhang, W., Kramer, E. M. and Davis, C. C. (2012). Similar genetic mechanisms underlie the parallel evolution of floral phenotypes. *PLoS ONE* 7, e36033. 10.1371/journal.pone.0036033 22558314 PMC3338646

[DEV203027C167] Zhang, W., Steinmann, V. W., Nikolov, L., Kramer, E. M. and Davis, C. C. (2013). Divergent genetic mechanisms underlie reversals to radial floral symmetry from diverse zygomorphic flowered ancestors. *Front. Plant Sci.* 4, 1-13. 23970887 10.3389/fpls.2013.00302 PMC3747361

[DEV203027C168] Zhang, W., Kramer, E. M. and Davis, C. C. (2016). Differential expression of CYC2 genes and the elaboration of floral morphologies in Hiptage, an old world genus of Malpighiaceae. *Int. J. Plant Sci.* 177, 551-558. 10.1086/687225

[DEV203027C169] Zhang, J., Stevens, P. F. and Zhang, W. (2017a). Evolution of floral zygomorphy in androecium and corolla in Solanaceae. *J. Syst. Evol.* 55, 581-590. 10.1111/jse.12275

[DEV203027C170] Zhang, T., Zhao, Y., Juntheikki, I., Mouhu, K., Broholm, S. K., Rijpkema, A. S., Kins, L., Lan, T., Albert, V. A. and Teeri, T. H. (2017b). Dissecting functions of SEPALLATA-like MADS box genes in patterning of the pseudanthial inflorescence of *Gerbera hybrida*. *New Phytol.* 216, 939-954. 10.1111/nph.14707 28742220

[DEV203027C171] Zhang, R., Fu, X., Zhao, C., Cheng, J., Liao, H., Wang, P., Yao, X., Duan, X., Yuan, Y., Xu, G. et al. (2020). Identification of the Key Regulatory Genes Involved in Elaborate Petal Development and Specialized Character Formation in *Nigella damascena* (Ranunculaceae). *Plant Cell* 32, 3095-3112. 10.1105/tpc.20.00330 32732312 PMC7534484

[DEV203027C172] Zhang, C. J., Rong, Y. L., Jiang, C. K., Guo, Y. P. and Rao, G. Y. (2022). Co-option of a carotenoid cleavage dioxygenase gene (CCD4a) into the floral symmetry gene regulatory network contributes to the polymorphic floral shape–color combinations in Chrysanthemum sensu lato. *New Phytol.* 236, 1197-1211. 10.1111/nph.18325 35719106

[DEV203027C174] Zhao, Y., Zhang, T., Broholm, S. K., Tähtiharju, S., Mouhu, K., Albert, V. A., Teeri, T. H. and Elomaa, P. (2016). Evolutionary co-option of floral meristem identity genes for patterning of the flower-like Asteraceae inflorescence. *Plant Physiol.* 172, 284-296. 27382139 10.1104/pp.16.00779 PMC5074612

[DEV203027C175] Zheng, X., Om, K., Stanton, K. A., Thomas, D., Cheng, P. A., Eggert, A., Simmons, E., Yuan, Y.-W., Conradi Smith, G. D. and Puzey, J. R. (2021). The regulatory network for petal anthocyanin pigmentation is shaped by the MYB5a/NEGAN transcription factor in Mimulus. *Genetics* 217, 355-385. 10.1093/genetics/iyaa036 PMC8045675 33724417

[DEV203027C176] Zhu, C., Bai, C., Sanahuja, G., Yuan, D., Farré, G., Naqvi, S., Shi, L., Capell, T. and Christou, P. (2010). The regulation of carotenoid pigmentation in flowers. *Arch. Biochem. Biophys.* 504, 132-141. 10.1016/j.abb.2010.07.028 20688043

[DEV203027C177] Zhu, W., Yang, L., Wu, D., Meng, Q., Deng, X., Huang, G., Zhang, J., Chen, X., Ferrándiz, C. and Liang, W. (2022). Rice SEPALLATA genes OsMADS5 and OsMADS34 cooperate to limit inflorescence branching by repressing the TERMINAL FLOWER1-like gene RCN4. *New Phytol.* 233, 1682-1700. 10.1111/nph.17855 34767634

