# Supplementary material for: Evolution and development of complex floral displays
Source: Development. 2024 Nov 5;151(21):dev203027. doi: 10.1242/dev.203027 (PMC11574353; doi:10.1242/dev.203027)
Supplement: Supplementary information [file develop-151-203027-s1.pdf]

Table S1. List of species discussed in the Review, detailing which section they are mentioned in. These species are categorized as exhibiting simple or more elaborate complexity, according to whether one or more of the developmental modules discussed in this Review are modified. A summary of which of the four modules is modified to produce the observed morphological diversity is included for each species. Abbreviations: I, inflorescence architecture; C, flower colour; O, organ identity; S, flower symmetry.

**Species**	**Order**	**Family**	**Level of complexity**	**Modules of complexity**
Antirrhinum majus	Lamiales	Plantaginaceae	Simple	C
Amorphophallus	Alismatales	Araceae	Elaborate	I, O, C
Anthriscus sylvestris	Apiales	Apiaceae	Elaborate	I, S
Apium graveolens	Apiales	Apiaceae	Simple	I
Aquilegia caerulea	Ranunculales	Ranunculaceae	Elaborate	O, C
Arabidopsis thaliana	Brassicales	Brassicaceae	Simple	I
Asclepias syriaca	Gentianales	Apocynaceae	Elaborate	I, O
Chirita	Lamiales	Gesneriaceae	Elaborate	S, C
Chrysanthemum	Asterales	Asteraceae	Elaborate	I, C
Clarkia gracilis	Myrtales	Onagraceae	Simple	C
Cornus	Cornales	Cornaceae	Elaborate	I, O
Daucus carota	Apiales	Apiaceae	Simple	I
Davidia	Cornales	Nyssaceae	Elaborate	I, O
Dietes robinsoniana	Asparagales	Iridaceae	Simple	O
Euphorbia rigida	Malpighiales	Euphorbiaceae	Elaborate	I, O, S
Gerbera hybrida	Asterales	Asteraceae	Elaborate	I, S, C
Gorteria diffusa	Asterales	Asteraceae	Elaborate	I, O, S, C
Helianthus annuus	Asterales	Asteraceae	Elaborate	I, S, C
Helleborus orientalis	Ranunculales	Ranunculaceae	Elaborate	O, C
Hibiscus trionum	Malvales	Malvaceae	Simple	C
Hydrangea	Cornales	Hydrangeaceae	Elaborate	I, O, C
Impatiens	Ericales	Balsaminaceae	Elaborate	I, O, C
Knautia macedonica	Dipsacales	Caprifoliaceae	Elaborate	I, S
Linaria spartea	Lamiales	Plantaginaceae	Elaborate	O, S
Mandragora caulescens	Solanales	Solanaceae	Simple	S
Marcgravia	Ericales	Marcgraviaceae	Elaborate	I, O
Meconopsis grandis	Ranunculales	Papaveraceae	Elaborate	O, C
Mimulus lewisii	Lamiales	Phrymaceae	Simple	C
Myosotis arvenis	Boraginales	Boraginaceae	Elaborate	I, C
Leucadendron discolor	Proteales	Proteaceae	Elaborate	I, O, C
Narcissus	Asparagales	Amaryllidaceae	Elaborate	O, C
Nicotiana obtusifolia	Solanales	Solanaceae	Simple	I
Nigella	Ranunculales	Ranunculaceae	Elaborate	O, C
Ophrys	Asparagales	Orchidaceae	Elaborate	O, C, S
Oryza sativa	Poales	Poaceae	Elaborate	I, O
Passiflora caerulea	Malpighiales	Passifloraceae	Elaborate	O, S, C

Development • Supplementary information

Petrea volubilis	Lamiales	Verbenaceae	Elaborate	O, S
Petunia hybrida	Solanales	Solanaceae	Elaborate	I, S, C
Pholidota imbricata	Asparagales	Orchidaceae	Simple	S
Schizanthus pinnatus	Solanales	Solanaceae	Elaborate	I, S, O, C
Senecio vulgaris	Asterales	Asteraceae	Elaborate	I, S
Silene latifolia	Caryophyllales	Caryophyllaceae	Elaborate	I, O
Syringa vulgaris	Lamiales	Oleaceae	Simple	I
Tagetes erecta	Asterales	Asteraceae	Elaborate	I, S, C
Tulipa spp.	Liliales	Liliaceae	Simple	O
Veronica gentianoides	Lamiales	Plantaginaceae	Elaborate	O, S, C

Development • Supplementary information
